# Bioprospecting the Metabolome of Plant *Urtica dioica* L.: A Fast Dereplication and Annotation Workflow in Plant Metabolomics

**DOI:** 10.1155/2022/3710791

**Published:** 2022-04-21

**Authors:** Keshab Bhattarai, Babita Paudel, Sujan Dahal, Parasmani Yadav, Niraj Aryal, Bikash Baral, Hari Datta Bhattarai

**Affiliations:** ^1^Center for Natural and Applied Sciences (CENAS), Kathmandu, Nepal; ^2^Institute of Biological Resources, Kathmandu, Nepal; ^3^Central Department of Botany, Tribhuvan University, Kirtipur, Kathmandu, Nepal

## Abstract

Plants have a pivotal role in ethnopharmacology, and their preparations are in use globally. However, getting down to the structure requires an effective workflow and mostly requires a time-consuming isolation process. Although bioassay-guided approaches are widely popular, they face a massive problem of rediscovery in recent times, especially in plant metabolomics. Mass spectrometry (MS)-based approach incorporated molecular networking via Global Natural Product Social Molecular Networking (GNPS) is considered here for the benefit of the fast screening of secondary metabolites. This study uses direct crude extracts obtained from various parts of the *Urtica dioica* plant for the characterization of secondary metabolites. The crude extract of the plant initially displayed promising antioxidant and anti-diabetic activities. Then, we employed mass spectrometry-based dereplication to identify the phytochemical components in the extracts. This led to the discovery of 7 unknown and 17 known secondary metabolites, which were further verified with the SIRIUS 4 platform, a computational tool for the annotation of compounds using tandem MS data. On the other hand, chasing the antioxidant activity of methanolic extract of *U. dioica* leaves, we employed a bioassay-guided isolation approach. With this method, we isolated and characterized compound **13**, a known molecule, which possessed strong antioxidant activity without showing much toxicity in the brine shrimp lethality test at the test concentration of 1 mg/mL. With our results, we advocate the MS-based approach as a good starting point for the dereplication of compounds from the complex crude extracts of plants.

## 1. Introduction

*Urtica dioica* L. has been in use for centuries in different parts of the world, especially in Asia and Europe, as food and herbal medicines [1–4]. To date, there are a few published scientific literatures about the utilization of *U. dioica* L. as an herbal medicine. In Nepal, as this plant possesses high therapeutic potential, these plants have been exploited for centuries by local tribes to cure different ailments, such as pain, inflammation, wound healing, and diabetes [5]. Several biological activities such as antimicrobial, antioxidant [6], hypoglycemic activity [7–10] of crude extract of *U. dioica* L., and other genera of *Urtica* have also been reported. Usage of various advanced techniques such as gas chromatography mass spectrometry (GC-MS) [11] and high-resolution mass spectrometry (HRMS) [12] has been performed for phytochemical profiling of this plant. A total of 35 new compounds have been reported from the plants belonging to the family Urticaceae in the period of 2000–2010 and among them, a new flavone has been mostly reported from *U. dioica* L. [13].

**Figure 1:**
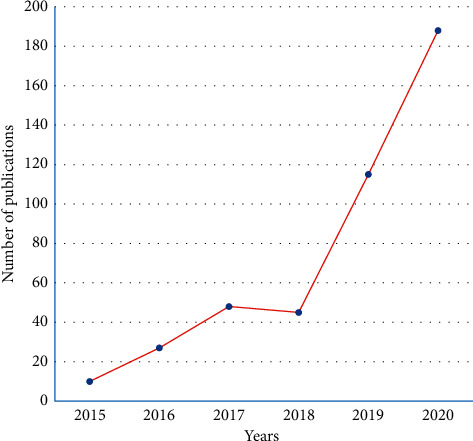
A graph showing article numbers published each year since 2016 involving GNPS for compound dereplication.

In regard to chemical analysis, mass spectrometry has been a recent favorite analytical tool also used for the analysis of natural products. Due to its high sensitivity and time advantage, it has broad applicability. HRMS data are utilized in various computational platforms to visualize and interpret the chemical repertoire that can be detected using MS [14]. In that context, Global Natural Product Social Molecular Networking (GNPS) has been widely popular to analyze MS2 raw data. With an increasing number of articles published each year (Figure 1), molecular networking-based dereplication has become one of the powerful tools for studying a diverse class of low molecular weight molecules in a single attempt [14]. Molecular networking works based on clustering structurally related metabolites having comparatively similar MS2 fragments. This allows instantaneous visual investigation of identical molecules, analogs, or compound classes. The networking system assembles mass spectrometric data by mining the spectral similarity between the available MS2 fragmentation patterns of structurally related metabolites. An individual node in the network corresponds to one consensus MS2 spectrum connected to other nodes with common fragmentation patterns of edges. Similarly, MS2 raw data can also be utilized in another *in silico* platform called SIRIUS 4, where the authors have claimed to provide annotation efficiency over 70% in the complex metabolomics data sets [15]. This technique has been successfully applied for the overall evaluation of untargeted and targeted secondary metabolites inhabited from diverse natural sources such as plants, animals, bacteria, fungi, and sponges [16–18]. This study has also relied on MS-based molecular networking to partially characterize compounds from methanolic extracts of leaves, stems, and roots of *U. dioica* L.

**Figure 2:**
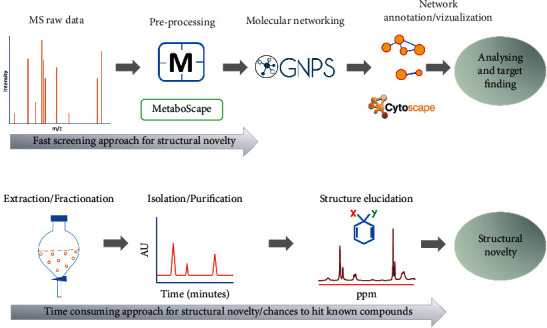
Metabolomics workflow showing the molecular networking approach and conventional isolation approach for finding structural novelty.

Here, we present the workflow (Figure 2), which begins with the acquisition of MS data and guides through the annotation of compounds in the crude extracts. While on the other hand, bioassay-guided isolation of the active compounds has been performed. The dereplication of relatively abundant known and unknown secondary metabolites has been performed to determine the plant species as a potential source of novel natural products.

## 2. Materials and Methods

### 2.1. Plant Collection

*U. dioica* L. was harvested from Kathmandu, Nepal (27.6863°N, 85.2949°E). The plant was authenticated by comparing the authentic voucher specimen deposited in Tribhuvan University Central Herbarium (TUCH) at Central Department of Botany, Tribhuvan University, Kirtipur, Kathmandu and deposited at the Center for Natural and Applied Sciences (CENAS), Kathmandu, Nepal, under the accession number UKIB_33.

### 2.2. Preparation of Crude Extracts

Air-dried plant samples leaves, stems, and roots (300 g each) were ground to powder and were extracted with a mixture of water and methanol (10 : 90). The filtrate was dried using a rotary evaporator. The obtained crude extract was dissolved in 200 mL of distilled water and extracted with 600 mL of hexane to remove nonpolar fatty compounds. The remaining water phase was further extracted with 600 mL of dichloromethane (DCM) to remove medium polar compounds. In addition, the remaining water phase was freeze-dried and dissolved in methanol. Thus, hexane, DCM, and methanol fractions were prepared.

### 2.3. *α*-Glucosidase Inhibition Assay

The *α*-glucosidase inhibitory activities were assayed following [19] protocol with slight modifications. An aliquot of 100 *μ*L of the test samples of various concentrations (0–100 *μ*g/mL) was mixed with 40 *μ*L of baker's yeast *α*-glucosidase preincubated at 37°C for 10 min. Then, the reaction was initiated with 1 mM of p-nitrophenyl-*α*-glucopyranoside (PNPG) in 50 mM phosphate buffer, pH 7.0, in a total volume of 1 mL. The reaction was terminated by adding 1.0 mL of 0.5 M Tris solution, and the absorbance of p-nitrophenol (PNP) released from PNPG was measured at 400 nm with a UV-visible spectrophotometer (Shimadzu, Japan). Reaction mixture without test samples was taken as negative control, and the reaction mixture with acarbose was employed as positive control.

### 2.4. Antioxidant Activity

Diphenylpicrylhydrazyl (DPPH) free radical scavenging activity of the samples was analyzed as described previously [20]. One mL of DPPH solution (0.1 mM of DPPH in methanol) was mixed with 3 mL of various concentrations (0–100 *μ*g/mL) of the test samples. The mixture was incubated at room temperature (RT) for 30 min, and the absorbance was measured at 517 nm in a UV-visible spectrophotometer. Reaction mixtures without the test sample and with BHA were used as negative and positive controls, respectively. The experiments were performed in triplicate.

### 2.5. Toxicity Assay

Brine shrimp lethality test was performed as described previously [21]. Briefly, the eggs of *Artemia salina* were hatched in aerated seawater equivalent in light at 25°C. The active larvae (*n* = 100) were selected and treated with various concentrations of test samples (0–1,000 *μ*g/mL). The effectiveness of the test samples was monitored after 24 h of treatment by observing the live larvae. The mortality rate of the larvae indicated the toxicity of test samples. Therefore, berberine chloride, a standard anticancer drug, was taken as a positive control, and brine shrimp larvae in salty water were taken as a negative control.

### 2.6. Bioactivity-guided Isolation of Antioxidant Compounds

The water fraction (1 g) of leaf extract was subjected to vacuum liquid chromatography using C_18_ODS column as the stationary phase and methanol–water mixture (0–100%) as a mobile phase. The fraction (280 mg) obtained at 30% of methanol in water showed strong DPPH reducing activity. For isolation of the active compound, the fraction was subjected to high-performance liquid chromatography (HPLC) using a C_18_ODS column (Phenomenex, size 250 mm × 10 mm, pore size 5 *μ*m) with the mobile phase as an acetonitrile–water mixture (2 mL/min). The solvent gradient was run as ACN/H_2_O (0.1% acetic acid); 0 min: 5/95, 25 min: 20/80, 28 min: 100/0, and continued up to 30 minutes. The isolated compound with an absorbance of 280 nm wavelength at 4.6 min displayed strong DPPH reducing activity. The purified compound was subjected ahead for structure elucidation.

### 2.7. Identification and Characterization of Compounds

The molecular structure of the purified compound was elucidated using spectroscopic techniques, such as nuclear magnetic resonance (NMR) spectroscopy (Bruker Avance III HD spectrometer; ^1^H: 400 MHz; ^13^C: 100 MHz, COSY, HSQC, and HMBC experiments in d_4_-methanol, 298K), and high-resolution mass spectrometry (HRMS). A 5 mm SMART probe head was used for NMR measurement of the purified compound. Once the structure was fully confirmed, the compound was searched in SciFinder, a database developed and offered by Chemical Abstracts Service (CAS). If the hit is found in the SciFinder, the compound is known, if not, then the compound is considered as new. Similarly, the molecular formulae for the compounds were initially predicted with Bruker Compass Data Analysis 4.0 software and further assured with SIRIUS 4 platform. These formulae were taken as input in SciFinder. Once the hits were not found within the database, these formulae of the respective compounds were also considered as unknown metabolites.

### 2.8. LC-HRMS Based Metabolomics

The samples of 0.2 mg/mL crude extract from the leaf, stem, and roots of the plant *U. dioica* in HPLC grade methanol were prepared to measure LC-HRMS. The data obtained from LC-HRMS were first analyzed in Bruker Compass DataAnalysis 4.0 software to assure the proper ionization of the compounds. Only the positively ionized HRMS data were processed through Bruker MetaboScape 3.0 software, omitting the poorly ionized negative mode data. The data were processed here for bucketing all molecular ions in a single file. Some specific parameters such as intensity threshold (counts); 5000, minimum peak length (spectra); 4, minimum peak length (recursive); 3, EIC correlation; 0.8 and mass calibration; true were optimized during bucketing. The adduct ions were confirmed [M +H ]^+^ as primary ion, [M + Na]^+^, [M + K]^+^, [2M + H]^+^, [2M + Na]^+^, [M + 2H]^2+^, [M + 2Na]^2+^ as seed ions and [M-H_2_O + H]^+^, [M + H_2_O + H]^+^, [2M + H_2_O + H]^+^, [2M-H_2_O + H]^+^ as common ions during processing. From the raw MS chromatogram, the retention time range of 4–45 minutes and molecular mass range of 200–1500 m/z were considered with the average MS2import method for generating the bucket list of diverse compounds adopting these parameters.

After bucketing, the software enlisted the molecular ions observed in the HRMS chromatogram of their related extract sources (leaves, root, and stem) into a column with their respective retention time, adduct ions or parent ions, molecular weight, and abundance (relative ion intensity). The bucket list of these molecular ions was exported in the mascot generic format (MGF) files, which was later unzipped into five different file formats, including Excel and GNPS file types. The GNPS file format was uploaded through the GNPS platform for molecular networking, where the GNPS web server clusters the tandem mass compounds in a molecular network based on minimum matched fragment ions; 5 and minimum cosine score; 0.7. The network data retrieved from the GNPS platform were analyzed and annotated via Cytoscape 3.8.2 [22]. Node and edge attributes were used, where nodes are displayed as a colorful pie chart to visualize the relative abundance of each ion in three sources of extracts (leaf, root, and stem). A single network was generated for the positive ionization modes using the GNPS 2 platform (Supplementary file S1). The list of known and putative unknown metabolites sorted by the relative abundance was generated from molecular networking. These compounds were additionally verified using CSI: FingerID analysis of individual raw MS data in SIRIUS 4 platform via molecular structure database search with *m/z* tolerance 20.

### 2.9. Statistical Analysis

All bioassay data are expressed as mean ± SD from a minimum of three biological replicates. In addition, 50% inhibition concentration (IC_50_) was calculated by linear regression analysis of the obtained data using Microsoft Excel 2016.

## 3. Results

### 3.1. Antidiabetic and Antioxidant Activities

Methanolic extract of the stem of *U. dioica* L. showed *a*-glucosidase inhibition activity in a concentration-dependent manner. However, the dichloromethane and hexane fractions did not show *α*-glucosidase inhibition activity at the test concentration range up to 100 *μ*g/mL. On the other side, the methanol fraction of *U. dioica* L. leaves displayed comparably strong DPPH scavenging activity (Table 1). This result indicated that *U. dioica* L. could be a potent source of antioxidants. Therefore, further work on the isolation and characterization of active compounds was performed using the bioassay-guided liquid chromatography technique.

**Figure 3:**
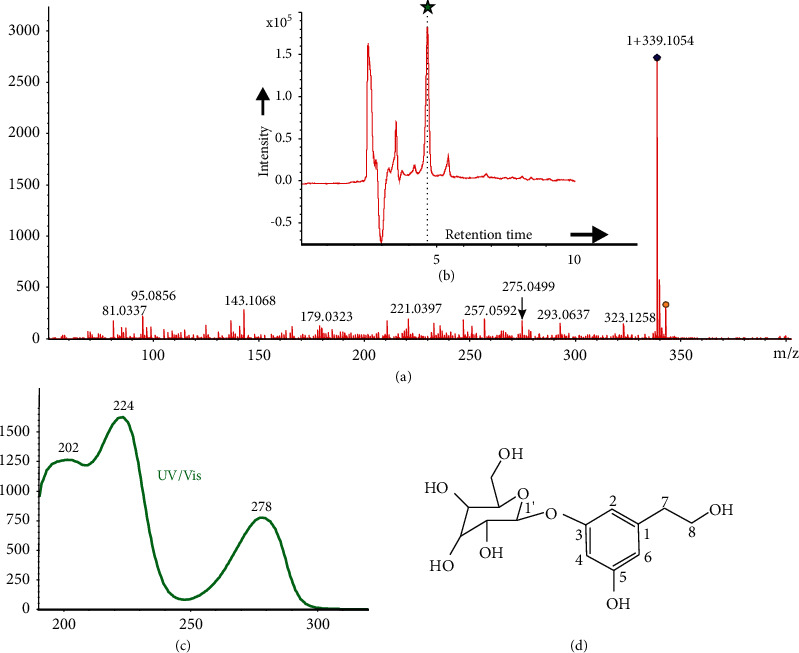
Structure of compound **13** showing chromatographic, spectroscopic, and spectrometric features. (*Note.* (a) MS2 ionization spectra from HRMS data, (b) UV chromatogram in HPLC system with eluted peak (R_T_ 4.6 min), (c) maximum UV absorbance curve, and (d) elucidated chemical structure based on NMR spectroscopy).

Compound **13** (5 mg) was obtained as a brownish-yellow gum. The high-resolution electrospray ionization mass spectrometry (HR-ESIMS) data of compound **13** shows a molecular weight of *m/z* 339.1054 [M + Na]^+^ with a molecular formula C_14_H_20_NaO_8_ and five degrees of unsaturation. The ^1^H NMR spectrum of compound **13** (Table 2, Supplementary Figures S27–S32) showed three aromatic proton signals of a metal substituted aromatic ring system and two coupled methylene signals. The ^13^C NMR spectrum showed the presence of a benzene ring along with one glucose molecule. In the HMBC spectrum, the key HMBC correlations of H-7 (*δ*H 2.60)/C-1 (*δ*C 130.5), C-2 (*δ*C 117.4), C-6 (*δ*C 123.2), confirmed that an ethoxyl group was linked to C-1 of the benzene ring as shown in Figure 3. In addition, an anomeric proton [*δ*H 4.95 (1H, d, J = 7.9 Hz)] and a characteristic proton signal of allose-H-3 at *δ*H 3.97 (1H, t, J = 2.8 Hz) were observed in ^1^H NMR spectrum, indicating the presence of an allopyranosyl unit. The site of allopyranosyl on the benzene ring of compound **13** was determined by the long-range HMBC correlation of H-1′ (*δ*H 4.95)/C-3 (*δ*C 145.4). Thus, the structure of compound **13** was depicted as 3, 5-dihydroxyphenethanol 3-O-*β*-D-allopyranoside. This compound was reported here for the first time from *U. dioica* L.

Compound **13** displayed antioxidant activity in terms of DPPH scavenging capacity. Its activity was higher than BHA (Table 1). However, it did not show antidiabetic activity and was nontoxic to brine shrimp at the test concentration of 100 *μ*g/mL. This indicates that the compound is nontoxic to eukaryotic cells.

### 3.2. Mass Spectrometry and Compound Annotation

Out of 12,046 molecular ions enlisted in the bucket list from MetaboScape, 2,859 (23.73%) molecular ions were found to have MS2 spectra, which were only reflected as nodes in the molecular network (Supplementary Figure S1). Among them, 1,481 molecular ions are self-looped nodes, 332 molecular ions formed 166 different clusters, each with holding two nodes, and the rest 1,039 molecular ions formed different bigger clusters. Among the ions in the network, the GNPS platform gave the hits of 228 molecular ions as known metabolites based on matching the GNPS library. In the present study, only seven unknown metabolites and 17 known metabolites have been reported based on their relative abundance (Tables 3 and 4). They were further assured with HRMS-based dereplication, CSI: FingerID via SIRIUS 4, and SciFinder as a computational approach for searching molecular structure databases.

**Figure 4:**
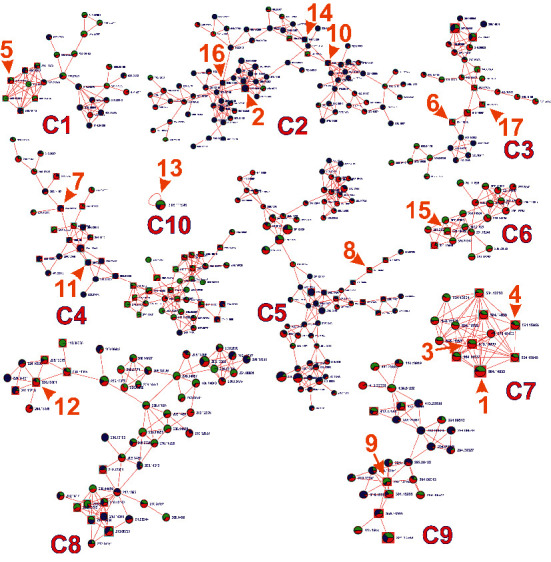
Molecular networking of MS2 data from *U. dioica* showing nine clusters including 17 known metabolites. (*Note.* Each node was identified by the neutral mass of the compound. The three colors green, red, and blue in each node represent the intensity of the compound detected in three different parts of the plant extract; leaf, stem, and root, respectively. The edge thickness in the network represents the similarity extent among the connected nodes).

Seventeen abundant compounds of known structural fingerprints (Supplementary Figure S2) in the plant extract were annotated individually based on MS2 data (Supplementary Figure, S3–S19) and fragmentation tree from SIRIUS 4. The MS2 key fragment ions with their annotated fragment motifs were analyzed thoroughly. HRMS data of compound **13** showed MS2 ionization from the precursor ion of sodium adduct [M + Na]^+^ and was clustered as self-looped node unlike other known clusters as shown in Figure 4.

Similarly, seven unknown metabolites annotated via MS2 fragmentation pattern comprised of four different clusters, as shown in Figure 5. These clusters selectively hold three peptides, one phenylalanine peptide, one fatty acyl glycoside, and two putative compounds (Table 4). These annotations were further assured by importing MS2 data to our SIRIUS 4 workflow (Supplementary Figures S20–S26 and S33–S39).

In the peptide class, all three members **1′**, **2′**, and **3′** compounds lose a common mass of 147 Da (C_9_H_9_NO), indicating a typical phenylalanine motif. Compounds **1′** and **2′** further lose 99 Da (C_5_H_9_NO), which possibly refers to valine as a fragment. Fragment ions at *m/z* 288.1709 and 328.1666 from compounds **1′** and **2′,** respectively, represent further loss of proline motif, while fragment ion m/z 316.1659 from compound **3′** indicates further loss of leucine or isoleucine motif and 97 Da (C_5_H_7_NO). The common fragment at *m/z* 259.1445 (C_15_H_19_N_2_O_2_) of all these compounds represents the characteristic fragment as a dipeptide motif, so here we conclude that the compounds might belong to the peptide class.

Compound **6′** gives a characteristic fragment at *m/z* 163.0603 (C_6_H_11_O_5_), possibly referring to a fructan monomer unit. The compounds **6′** and **7′** have a common fragment at *m/z* 69.0335 (C_4_H_5_O), indicating a furan ring as a stable modified arrangement of a fructan monomer. The rest of the fragments seem ambiguous, suggesting the compounds having an unknown structure.

In the phenylalanine-peptide class, compound **5′** shows a strong fragment at *m/z* 445.1761 with continuous loss of ammonia (17 Da) and carbonyl (28 Da) motifs. The fragment further loses carbonyl and water units continuously to yield stable fragments at *m/z* 417.1812 and 399.1709, respectively. The mutual fragments at *m/z* 462.2030, 445.1761, and 427.1655 lose 147 Da from their individual skeletons to give stable fragments at *m/z* 315.1342, 298.1078, and 280.0973, respectively, which is the crucial evidence of phenylalanine embedded in the structure. Another stable fragment at *m/z* 269.1287 arises from the original molecule after the successive loss of three carbonyl motifs, one ammonia, one water, and other subunits, which possibly indicate at least two peptide bonds present in the original compound. This classified the compounds possibly belonging to the phenylalanine peptide family.

In the compound class of fatty acyl glycosides, the stable fragment at *m/z* 209.1539 refers to the polyketide skeleton (C_13_H_21_O_2_) with 4-ring double bond equivalence. This polyketide fragment further loses the water units, giving successive fragments at *m/z* 191.1437 (C_13_H_17_O). The fragment resembled dehydrated glucose at *m*/*z* 163.0603 (C6H10O5), with further loss two water subunits one after another to yield stable fragments at *m/z* 145.0499 (C_6_H_9_O_4_) and 127.0391 (C_6_H_7_O_3_), respectively. These fragmentation patterns indicate the compound-bearing polyketide chain and glycosyl unit, comprising the family fatty acyl glycosides.

## 4. Discussion

Two major methods for the dereplication of bioactive secondary metabolites have been emphasized independently in this work from the plant *U. dioica* L. Among these two, the bioactivity-guided approach is a conventional method and was found to be time-consuming. The isolation of compound **13** was done with a bioassay-guided isolation approach. Upon further analysis, its structure was found to be reported earlier from *Centaurea urvillei* DC. subsp. *urvillei* with anticancer activity against breast cancer cell lines [36] and *Veronica thymoides* subsp. *pseudocinerea* [40] with antioxidant activity. However, compound **13** was reported for the first time from the genus *Urtica*. The isolation of other compounds from bioactive fractions was not achievable in our study, perhaps they were low UV active or lost to column during fractionation. So it is not always the case in bioassay-guided isolation that every bioactive component is achievable with the limited technical resources.

While, on the other hand, the HRMS dereplication approach combined with molecular networking predicts possible structural hits within a short time, allowing someone to prioritize the crude fractions from very early on. This priority could be based on structural novelty, compound size, and abundance. This can save us more time and resources from the repetitive isolation of known chemical entities. Although NMR-based structure elucidation is the gold standard in NP chemistry, there are still limitations to this technique. Here also, compound **13** was already predicted as 3,5-dihydroxyphenethanol-3-O-D-allopyranoside via SIRIUS 4 platform and later confirmed via spectroscopic analysis after extensive isolation.

In our study, we also find that the quality of MS2 data is key for structural fingerprint findings. These fingerprints are utilized by publicly available tools like SIRIUS 4. Our study is completely in line with the author's claims. However, more advanced algorithms and timely upgrades for such tools are key to improvement. In the past, this platform has been used generally for annotating the molecular formula only [41,42]. In this instance, we used its embedded CSI: FingerID and CANOPUS tools to predict compound structure from several databases, obligating the Natural Products database [43]. Some of the compounds, such as compounds **9**, **10**, **11**, **13**, **14**, **15,** and **16** have no match with GNPS spectral library database but with the Natural Product database embedded with SIRIUS 4. The rest of the compounds were verified with their GNPS spectrum ID (Table 3). The lack of GNPS spectral matching for these compounds might be due to the misrepresentation of precursor ions. For instance, compound **13** has the precursor ion as [M + Na]^+^ with m/z 339.1054, although the GNPS recognized it as [M + H]^+^ ion and mislead the spectral match. Compound **4** has also matched to Massbank spectral datasets given to their Massbank access number (Table 3). This further supports the annotation made by GNPS and SIRIUS 4.

In terms of activity, the strong *α*-glucosidase inhibition activity of methanolic stem extract can be justified as we annotated vicenin 2, roseoside, and Quercetin 3-(2G-xylosylrutinoside) in abundance that are already known for their strong antidiabetic properties [29, 30, 44]. However, to thoroughly verify this assumption and these compounds, one needs to isolate them and confirm their structure by spectroscopic analysis. In addition of that, the further isolation, characterization, and bioassay of those seven unknown compounds are worthy to carry out.

## 5. Conclusion

*U. dioica* L. is considered a traditionally important plant in Nepalese culture. It carries medicinal importance and has been consumed at the local level in the form of vegetables as well. It is prioritized due to its antidiabetic activity. Our study has successfully annotated compounds that hold *α*-glucosidase activity, which ultimately supports these community-based claims. Moreover, this plant is associated with cosmetic abilities, which were also verified in our study. Further work to isolate new annotated compounds is still necessary. However, our work sets up a vital chemical foundation for this plant.

## Figures and Tables

**Figure 5:**
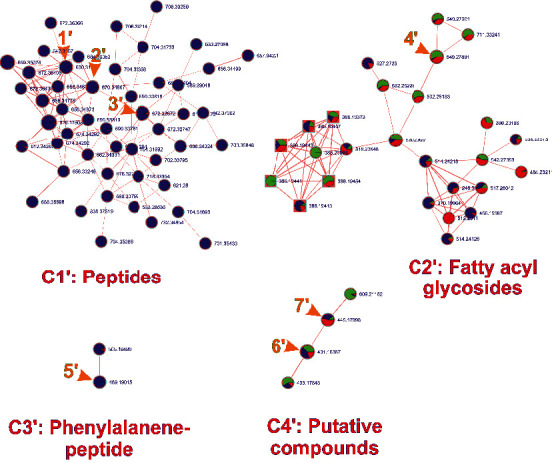
Molecular networking of MS2 data from *U. dioica* showing four clusters with seven unknown metabolites. (*Note.* Each node was identified by the neutral mass of the compound. The ratio of three colors as green, red, and blue in each node represents the relative intensity of the compound detected in three different parts of the plant extract; leaf, stem, and root, respectively. The edge thickness in the network represents the similarity extent among the connected nodes).

**Table 1:** *α*-Glucosidase inhibition, DPPH free radical scavenging, and brine shrimp toxicity activities of *U. dioica* L.

Test samples	*α*-glucosidase inhibition (IC_50_) (*µ*g/mL)				DPPH free radical inhibition (IC_50_) (*µ*g/mL)		Brine shrimp toxicity (1 mg/mL)
	ME	DCME	HE	ME	DCME	HE	ME
*U. dioica* L. (stem)	62.5 ± 5.2	NA	NA	38.9 ± 1.9	NA	NT	7%
*U. dioica* L. (root)	NA	NA	NA	12.1 ± 0.6	NA	NT	8%
*U. dioica* L. (leaf)	NA	NA	NA	11 ± 1.2	26.3 ± 1.4	NT	7%
Acarbose (+ve standard)	50 ± 3.6			NT	NT	NT	NT
Potassium dichromate	NT			NT			100%
Compound **13** from *U. dioica* L. leaf	NA			4.5 ± 0.2			NA
BHA	NT			4.8 ± 0.2			NT

Mean ± SD; ME, methanol extract; DCME, dichloromethane extract; HE, hexane extract; IC_50_, 50% inhibition of activity; NA, no activity; NT, not tested, BHA, Butylatedhydroxynisole.

**Table 2:** NMR spectroscopic data of compound 13 (^1^H: 400 MHz; ^13^C: 100 MHz, in CD_3_OD).

	*δ*C	*δ*H (*J* in hz)	HMBC	COSY
1	132.2	—	—	—
2	119.9	7.07 (d, 1.3)	39.5, 132.8, 125.2, 146.7	—
3	146.7	—	—	—
4	116.9	6.81 (d, 1.0)	146.7, 146.6, 119.9	—
5	146.6	—	—	—
6	125.2	6.77 (d, 1.0)	146.6, 119.9, 132.8	—
7	39.5	2.69 (t, 7.3)	132.8, 62.5, 132.8, 125.2, 119.9	3.92
8	62.5	3.92 (t, 7.3)	39.5, 132.8	2.69
1′	104.4	4.78 (d, 7.9)	146.7, 77.7	3.47
2′	77.7	3.47 (dd, 7.9, 2.8)	104.4, 74.9	4.78
3′	74.9	3.47 (t, 2.8)	77.7, 78.4	—
4′	78.4	3.35 (dd, 9.8, 2.8)	74.9, 71.4	—
5′	71.4	3.47	78.4, 64.3	—
6′	64.3	3.74	71.4, 78.4	
		3.46		

**Table 3:** Predicted known metabolites annotated and dereplicated with tandem MS data from *U. dioica* L.

Comp No.	^12^ C m/z	Parent ion	Molecular formula	Error ppm	R_T_ (min)	Proposed structure	Annotation^#^ (spectral data source)<	Total ions intensity	Leaf ext. int.	Root ext. int.	Stem ext. int.	Ref.
1	565.1553	[M+H]^+^	C_26_H_28_O_14_	0.1	13.8	Vicenin 3	CCMSLIB00004696062^*∗*^	3360987	1383074	274552	1703361	[23]
2	360.1078	[M+H]^+^	C_18_H_17_NO_7_	0.1	15.2	Clovamide	CCMSLIB00000845552^*∗*^	3241983	23828	2980171	237984	[24]
3	579.1716	[M+H]^+^	C_27_H_30_O_14_	1.4	14.8	Violanthin	CCMSLIB00004695807^*∗*^	1228234	519225	52679	656330	[25]
4	595.1668	[M+H]^+^	C_27_H_30_O_15_	1.7	12.7	Vicenin 2	CCMSLIB00005745586^*∗*^, PR303371^*λ*^, PR310993^*λ*^	1037798	375851	80058	581889	[26]
5	387.2019	[M+H]^+^	C_19_H_30_O_8_	1.4	13.6	Roseoside	CCMSLIB00000851547^*∗*^	935573	330113	261540	343920	[27]
6	595.1662	[M+H]^+^	C_27_H_30_O_15_	0.8	16.6	Nicotifolin	CCMSLIB00000846155^*∗*^	886166	205035	28100	653031	[28]
7	265.1434	[M+H]^+^	C_15_H_20_O_4_	0.0	14.6	Curcuminol G	CCMSLIB00004715428^*∗*^	854626	8106	606021	240499	[29, 30]
8	279.1707	[M+H]^+^	C_15_H_22_N_2_O_3_	1.4	19.6	Phe-leu	CCMSLIB00003134492^*∗*^	646071	202418	48413	395240	[31]
9	263.1281 285.1335	[M+H]^+^, [M+Na]^+^	C_15_H_18_O_4_	1.1	15.5	Austricin	—	544856	327953	58519	158384	[32]
10	343.1179	[M+H]^+^	C_19_H_18_O_6_	0.7	15.6	Tetramethoxy flavone	—	521797	2555	423546	95696	[33]
11	285.2215	[M+H]^+^	C_20_H_28_O	0.7	18.3	Retinene	—	478968	10869	456532	11567	[34]
12	227.1281	[M+H]^+^	C_12_H_18_O_4_	1.2	15.9	12-Hydroxyjasmonic acid	CCMSLIB00000856124^*∗*^	390475	103512	53281	233682	[35]
13	339.1054	[M+Na]^+^	C_14_H_20_O_8_	1.7	9.9	3, 5-dihydroxyphenethanol 3-O-*β*-D	—	318849	160121	63498	95230	[36]
14	341.1384	[M+H]^+^	C_20_H_20_O_5_	0.1	15.1	Flavaprenin	—	313196	10593	284531	18072	[37]
15	322.2018	[M+H]^+^	C_18_H_27_NO_4_	1.5	15.8	O-hydroxycapsaicin	—	256745	150679	4759	101307	[38]
16	536.1762	[M+H]^+^	C_25_H_29_NO_12_	0.0	15.0	Davallioside A	—	205433	0	181536	23897	[39]
17	743.2035	[M+H]^+^	C_32_H_38_O_20_	0.8	14.2	Quercetin 3-(2G-xylosylrutinoside)	CCMSLIB00000845960^*∗*^	149503	30820	7076	111607	[30]

^*∗*^GNPS library spectrum ID, **#** SIRIUS 4 verified, *λ* Massbank spectral access.

**Table 4:** Predicted novel compounds annotated and dereplicated with tandem MS data from *U. dioica* L.

Comp No.	^12^ C m/z	Parent ion	Molecular formula	Error ppm	R_T_ (min)	Predicted compound class	CSI: FingerID (score)	Relative ions intensity (leaf_stem_root)	Abundance priority
1	631.3237	[M+H]^+^	C_34_H_42_N_6_O_6_	0.0	17.2	Putative peptide	69.75%	0.002_0.004_0.994 Cluster-1′	Root

2	671.3555	[M+H]^+^	C_37_H_46_N_6_O_6_	1.5	21.6	Putative peptide	56.69%	0.004_0.008_0.989 Cluster-1′	Root

3	673.3709	[M+H]^+^	C_37_H_48_N_6_O_6_	0.2	20.0	Putative peptide	61.42%	0.557_0.400_0.042 Cluster-1′	Leaf and stem

4	550.2863	[M+H]^+^	C_25_H_43_NO_12_	0.9	15.1	Fatty acyl glycoside	—	0.558_0.407_0.035 Cluster-2′	Leaf and stem

5	490.1973	[M+H]^+^	C_27_H_27_N_3_O_6_	0.2	13.7	Phenylalanine-peptide derivative	50.68%	0.004_0.006_0.990 Cluster-3′	Root

6	432.1714	[M+H]^+^	C_15_H_29_NO_13_	3.3	5.0	Putative compound	—	0.373_0.203_0.424 Cluster-4′	Leaf, stem, and root

7	446.1874	[M+H]^+^	C_16_H_31_NO_13_	1.5	5.1	Putative compound	51.79%	0.096_0.545_0.359 Cluster 4′	Stem and root

## Data Availability

We provide supporting and necessary data for the publication of the article. All supporting data are presented in the article and additional files. The sample voucher specimen is deposited in the plant herbarium of Tribhuvan University and can be accessed upon reasonable request.

## Abbreviations

BHA: Butylated hydroxyanisole
DPPH: 2,2-diphenyl-1-picrylhydrazyl
GNPS: Global natural product social molecular networking
HPLC: High-performance liquid chromatography
HRMS: High-resolution mass spectrometry
NMR: Nuclear magnetic resonance
NP: Natural Product.
