# Supplementary material for: Bioprospecting the Metabolome of Plant Urtica dioica L.: A Fast Dereplication and Annotation Workflow in Plant Metabolomics
Source: Evid Based Complement Alternat Med. 2022 Apr 21;2022:3710791. doi: 10.1155/2022/3710791 (PMC9050285; doi:10.1155/2022/3710791)

Title: Bioprospecting the metabolome of plant *Urtica dioica* L.: A fast dereplication and annotation workflow in plant metabolomics

Keshab Bhattarai[1,2,α], Babita Paudel[1,α], Sujan Dahal[3], Parasmani Yadav[3], Niraj Aryal[1], Bikash Baral[2], Hari Datta Bhattarai[1,3*]

[1]Center for Natural and Applied Resources (CENAS), Kathmandu, Nepal

[2]Institute of Biological Resources, Kathmandu, Nepal

[3]Central Department of Botany, Tribhuvan University, Kirtipur, Kathmandu, Nepal

[α]Authors have equal contributions.

[*]Corresponding author, HD Bhattarai haridatta.bhattarai@cdb.tu.edu.np

Supplementary Informations

Table of Contents: **Page number**

**Fig. S1.** Molecular networking of all compounds	3
**Fig. S2.** Chemical scaffolds of known compounds	4
**Fig. S3. Compound 1**, m/z 565.1553 $[M+H]^+$, 13.8 mins	5
**Fig. S4. Compound 2**, m/z 360.1078 $[M+H]^+$, 15.2 mins	5
**Fig. S5. Compound 3**, m/z 579.1716 $[M+H]^+$, 14.8 mins	6
**Fig. S6. Compound 4**, m/z 595.1668 $[M+H]^+$, 12.7 mins	6
**Fig. S7. Compound 5**, m/z 387.2019 $[M+H]^+$, 13.6 mins	7
**Fig. S8. Compound 6**, m/z 595.1662 $[M+H]^+$, 16.6 mins	7
**Fig. S9. Compound 7**, m/z 265.1434 $[M+H]^+$, 14.6 mins	8
**Fig. S10. Compound 8**, m/z 279.1707 $[M+H]^+$, 19.6 mins	8
**Fig. S11. Compound 9**, m/z 263.1281 $[M+H]^+$ and 285.1335 $[M+Na]^+$, 15.5 mins	9
**Fig. S12. Compound 10**, m/z 343.1179 $[M+H]^+$, 15.6 mins	9
**Fig. S13. Compound 11**, m/z 285.2215 $[M+H]^+$, 18.3 mins	10
**Fig. S14. Compound 12**, m/z 227.1281 $[M+H]^+$, 15.9mins	10
**Fig. S15. Compound 13**, m/z 339.1054 $[M+Na]^+$, 9.9 mins	11
**Fig. S16. Compound 14**, m/z 341.1384 $[M+H]^+$, 15.1 mins	12
**Fig. S17. Compound 15**, 322.2018 $[M+H]^+$, 15.8 mins	12
**Fig. S18. Compound 16**, 536.1762 $[M+H]^+$, 15.0 mins	13
**Fig. S19. Compound 17**, 743.2035 $[M+H]^+$, 14.2 mins	13
**Fig. S20. Compound 1'**, m/z 631.3237 $[M+H]^+$, 17.2 mins	14
**Fig. S21. Compound 2'**, m/z 671.3555 $[M+H]^+$, 21.6 mins	14
**Fig. S22. Compound 3'**, m/z 673.3709 $[M+H]^+$, 20.0 mins	15
**Fig. S23. Compound 4'**, m/z 550.2863 $[M+H]^+$, 15.1 mins	15
**Fig. S24. Compound 5'**, m/z 490.1973 $[M+H]^+$, 13.7 mins	16
**Fig. S25. Compound 6'**, m/z 432.1713 $[M+H]^+$, 5.0 mins	16
**Fig. S26. Compound 7'**, m/z 446.1874 $[M+H]^+$, 5.1 mins	17
**Fig. S27.** Spectra 1: HNMR of compound 13	18
**Fig. S28.** Spectra 2: ^{13}CNMR of compound 13	18
**Fig. S29.** Spectra 3: DEPT-135 of compound 13	19
**Fig. S30.** Spectra 4: COSY of compound 13	19
**Fig. S31.** Spectra 5: HSQC of compound 13	20
**Fig. S32.** Spectra 6: HMBC of compound 13	20
**Fig. S33.** Fragmentation tree of compound 1'	21
**Fig. S34.** Fragmentation tree of compound 2'	22
**Fig. S35.** Fragmentation tree of compound 3'	23
**Fig. S36.** Fragmentation tree of compound 4'	24
**Fig. S37.** Fragmentation tree of compound 5'	25
**Fig. S38.** Fragmentation tree of compound 6'	26
**Fig. S39.** Fragmentation tree of compound 7'	26

Fig. S1. Molecular networking of all compounds

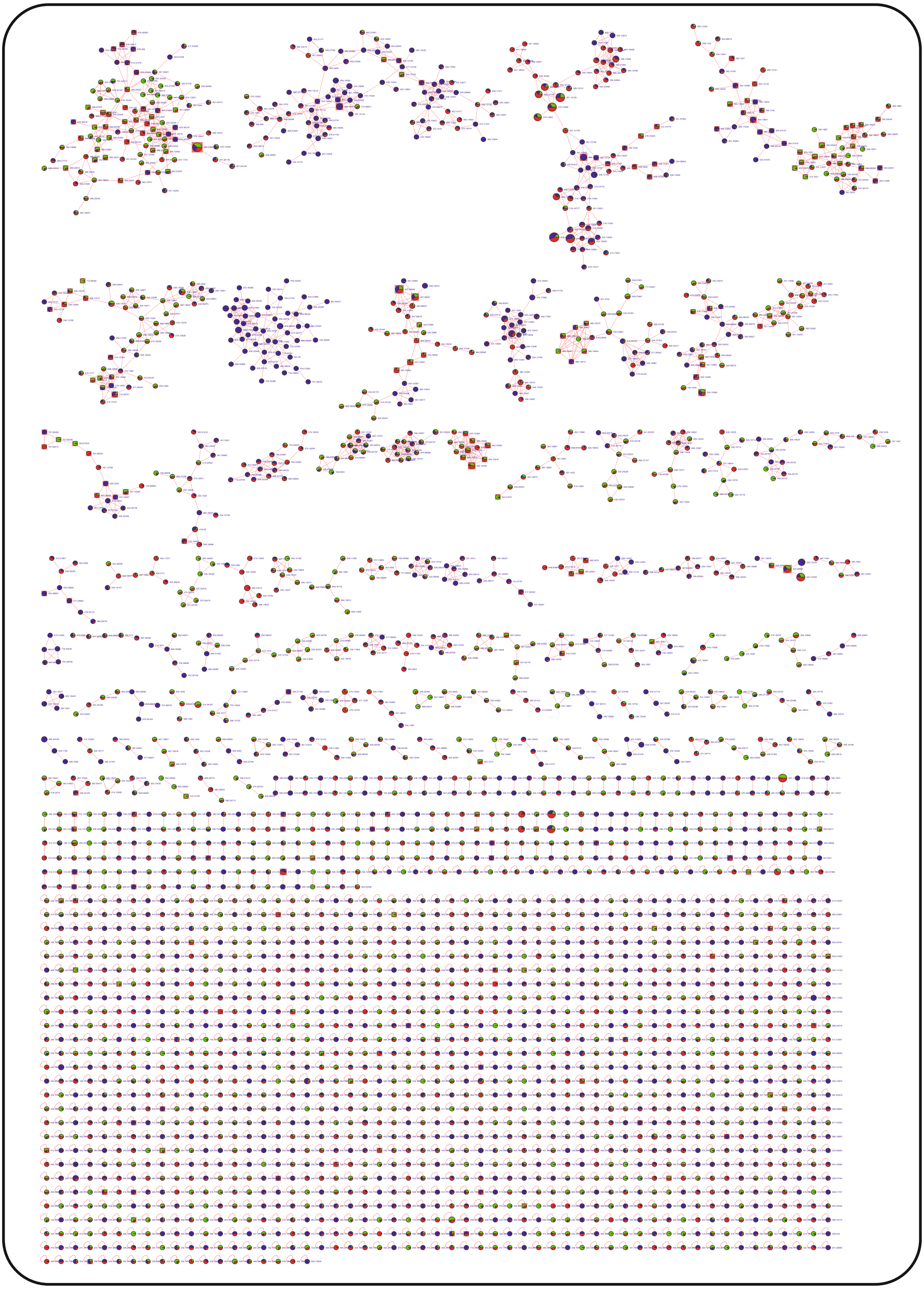

Fig. S2. Chemical scaffolds of known compounds

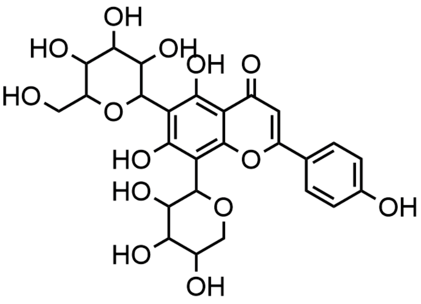

Vicenin 3

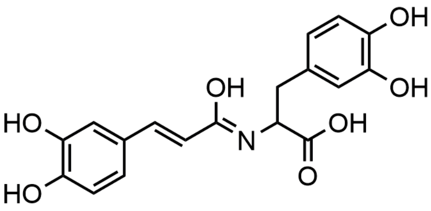

Clovamide

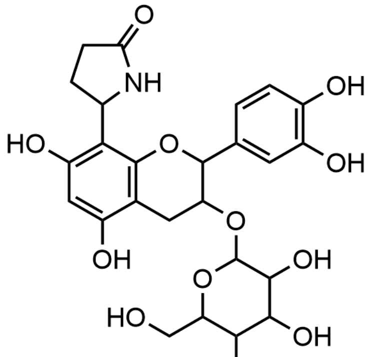

Davallioside A

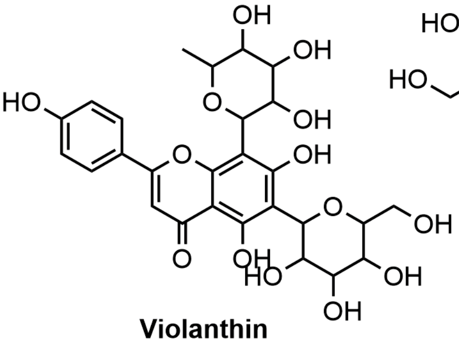

Violanthin

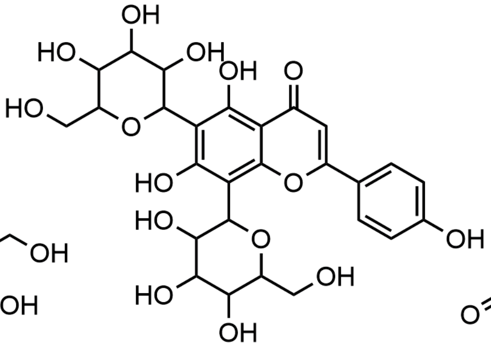

Vicenin 2

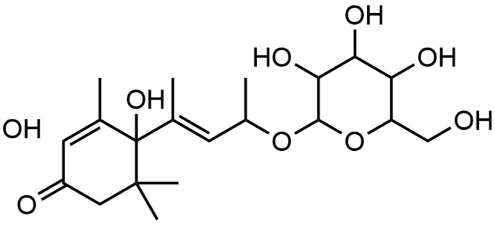

Roseoside

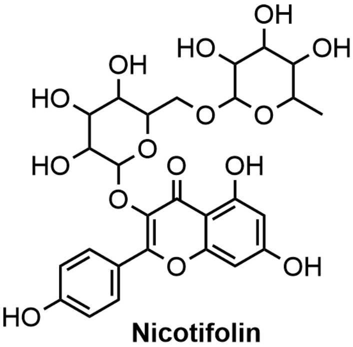

Nicotifolin

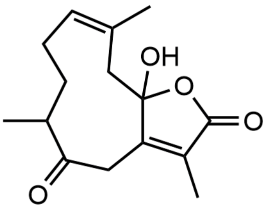

Curcuminol G

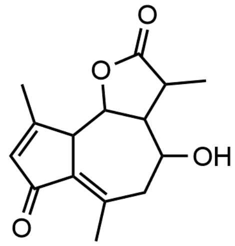

Austricin

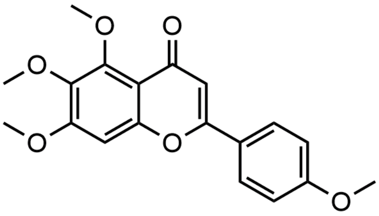

Tetramethoxyflavone

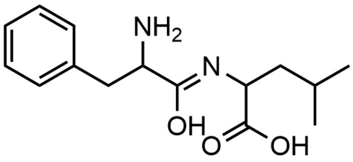

L-Phenylalanyl-L-leucine

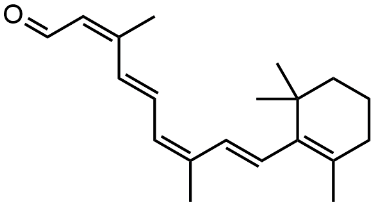

Retinene

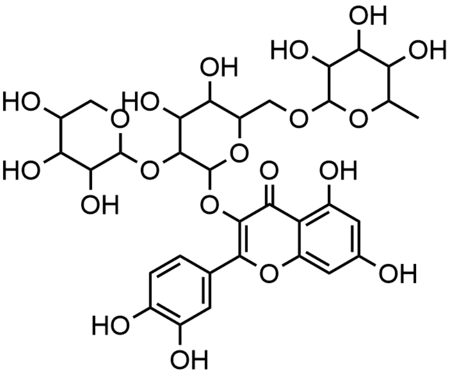

Quercetin 3-(2G-xylosylrutinoside)

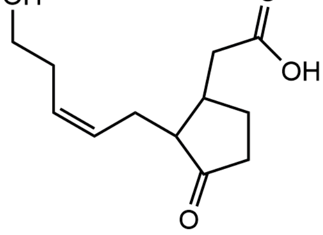

12-Hydroxyjasmonic acid

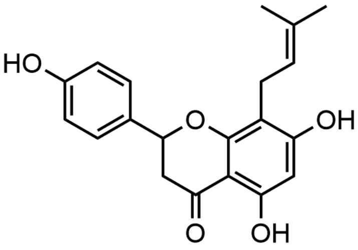

Flavaprenin or 8-prenylnaringenin

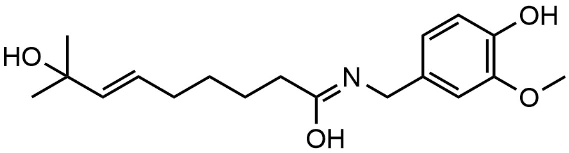

o-hydroxycapsaicin

Fig. S3

Compound 1, m/z 565.1553 $[M+H]^+$, 13.8 mins

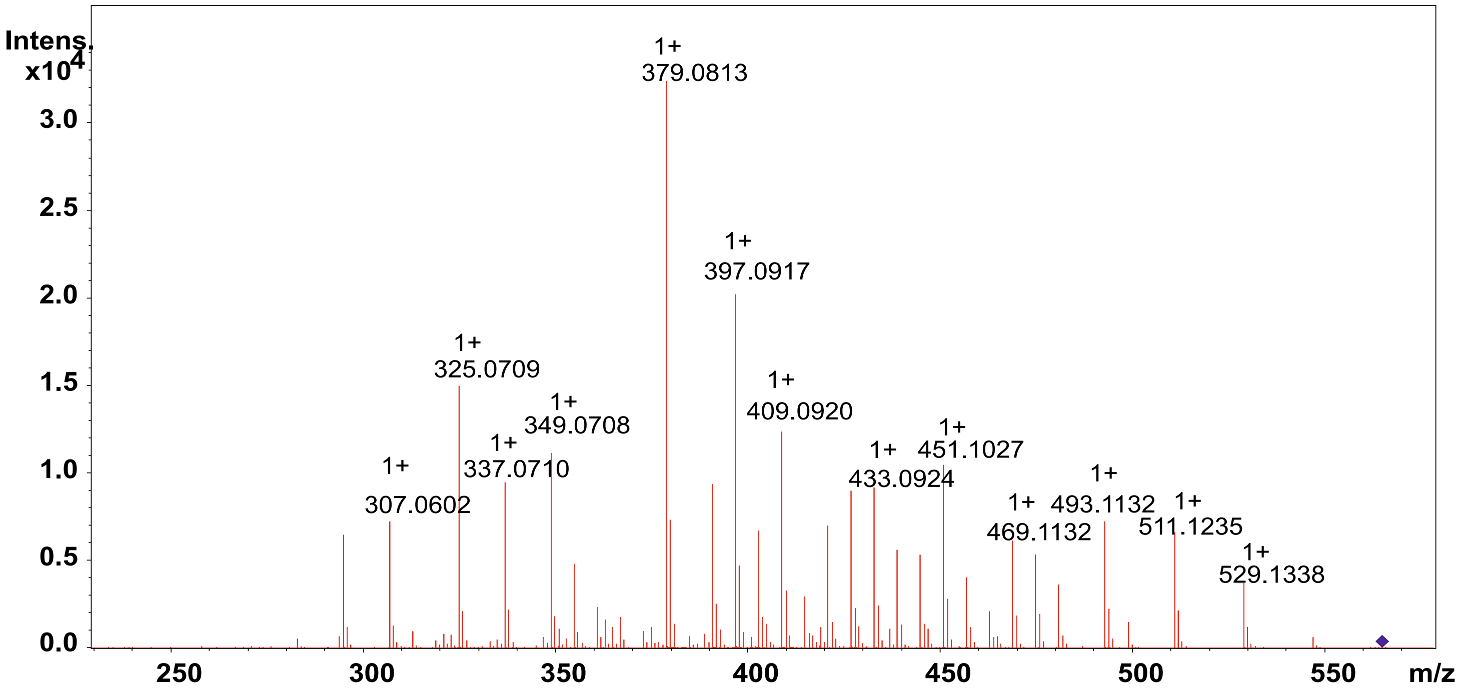

Fig. S4

Compound 2, m/z 360.1078 $[M+H]^+$, 15.2 mins

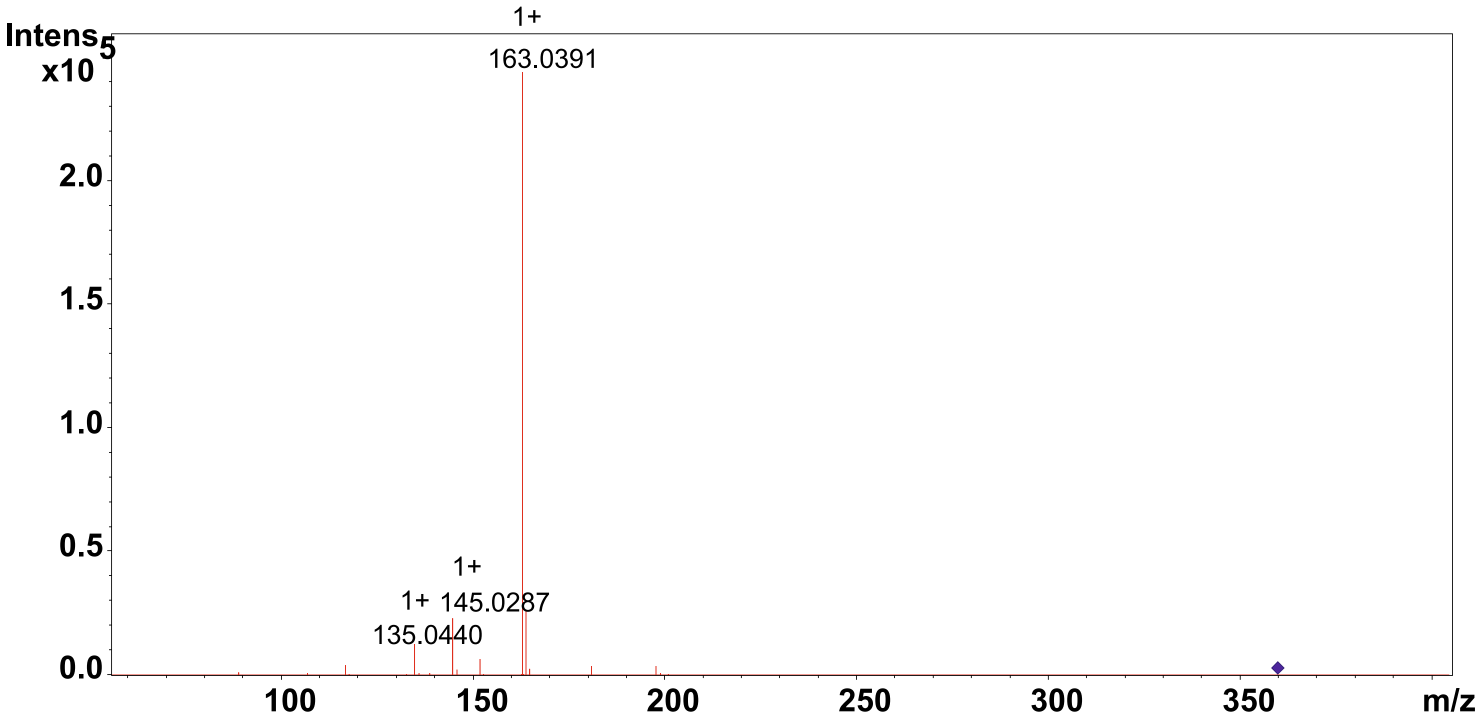

Fig. S5

Compound 3, m/z 579.1716 $[M+H]^+$, 14.8 mins

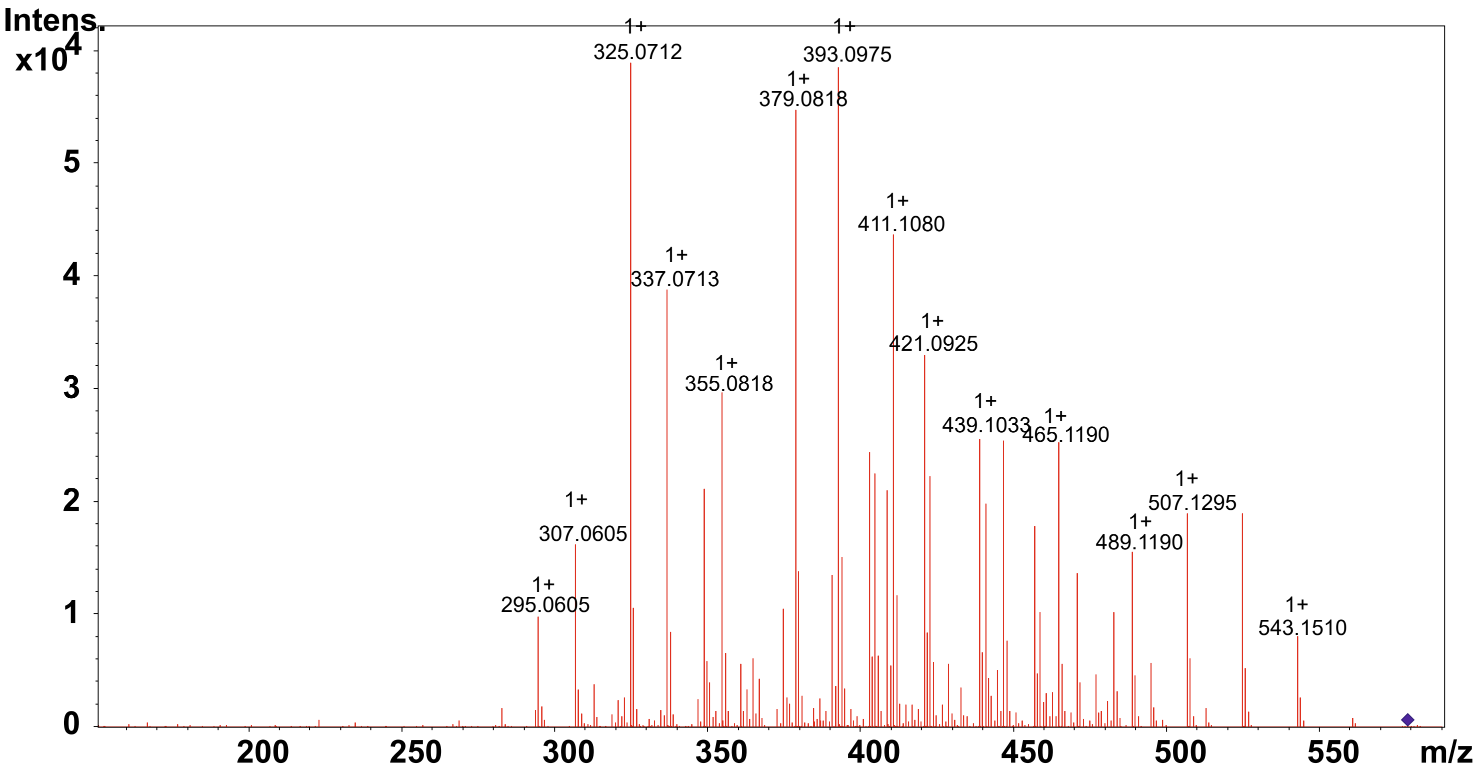

Fig. S6

Compound 4, m/z 595.1668 $[M+H]^+$, 12.7 mins

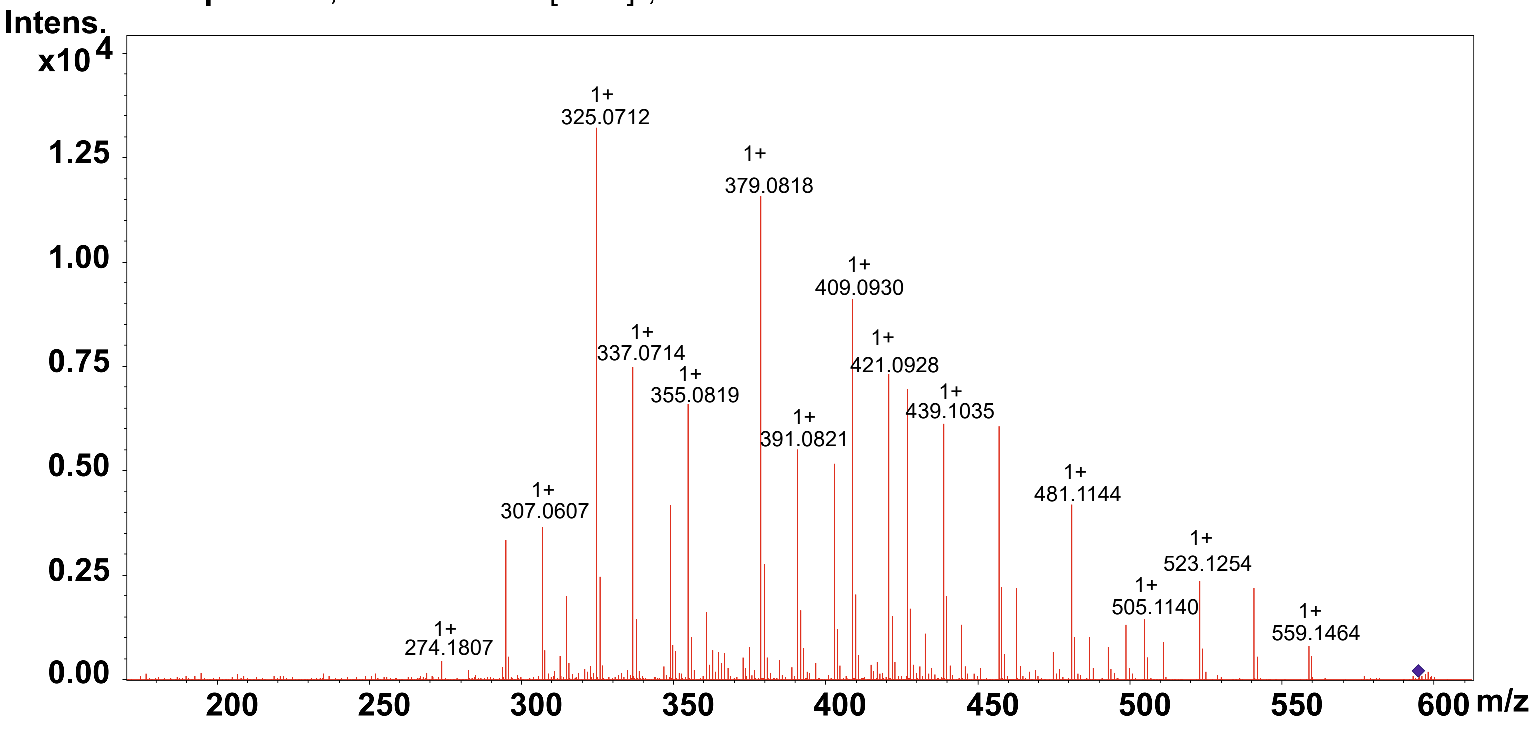

Fig. S7

Compound 5, m/z 387.2019 $[M+H]^+$, 13.6 mins

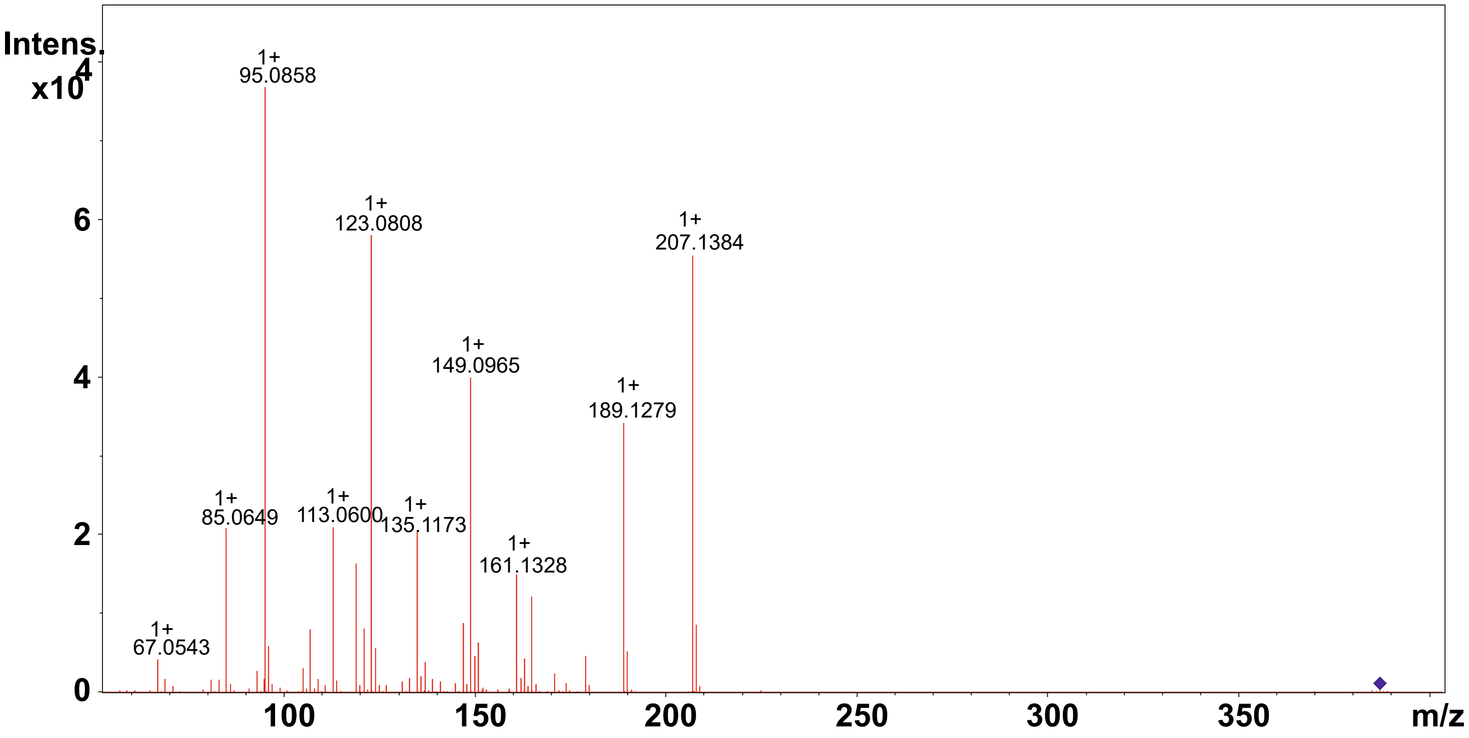

Fig. S8

Compound 6, m/z 595.1662 $[M+H]^+$, 16.6 mins

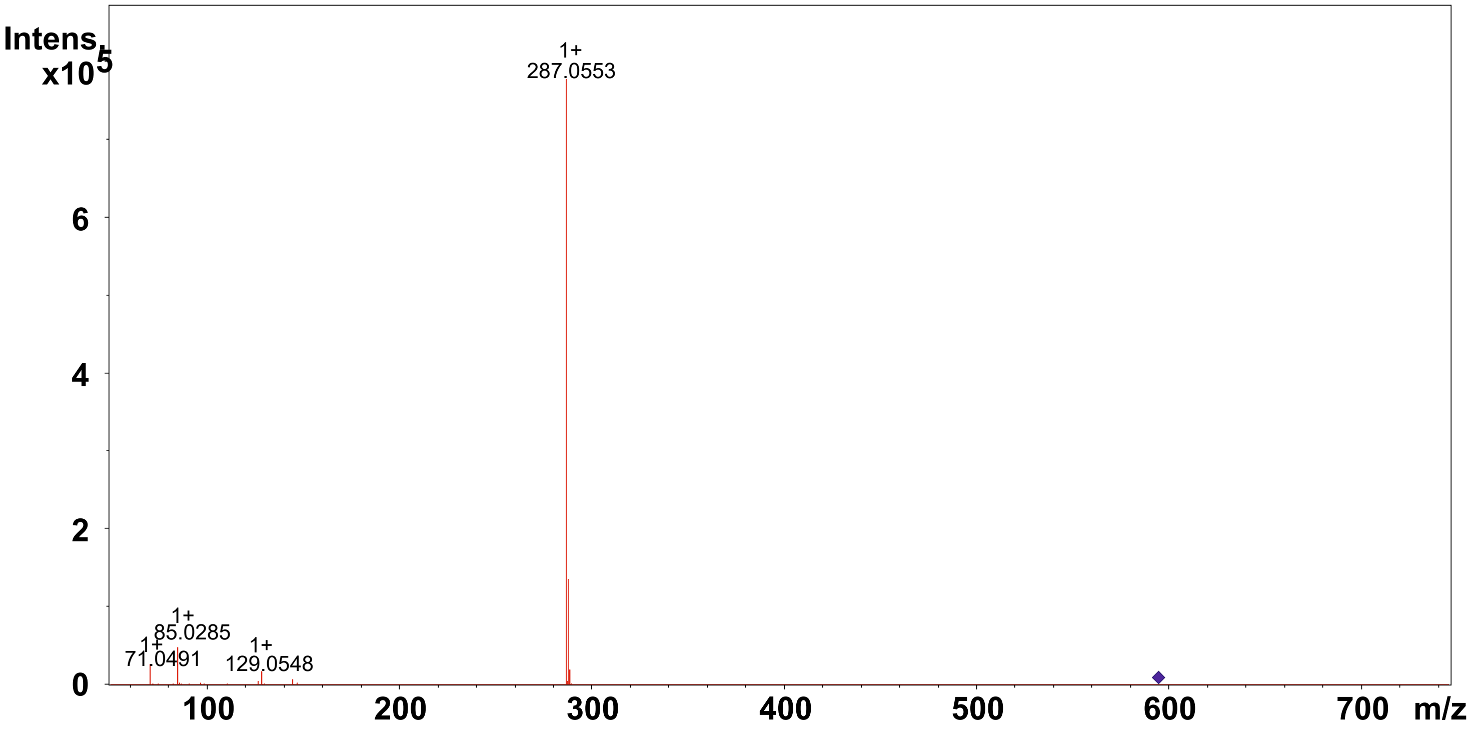

Fig. S9

Compound 7, m/z 265.1434 [M+H]$^+$, 14.6 mins

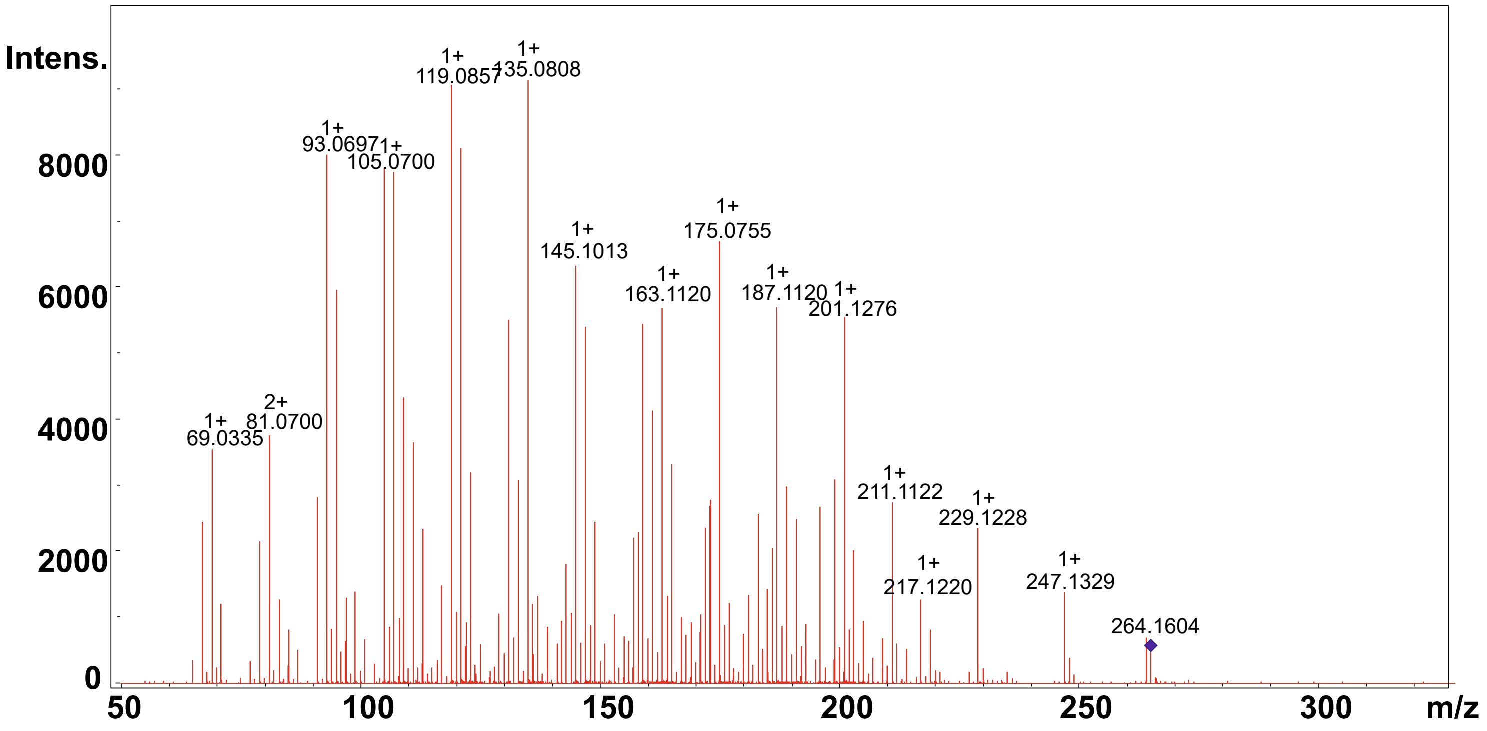

Fig. S10

Compound 8, m/z 279.1707 [M+H]$^+$, 19.6 mins

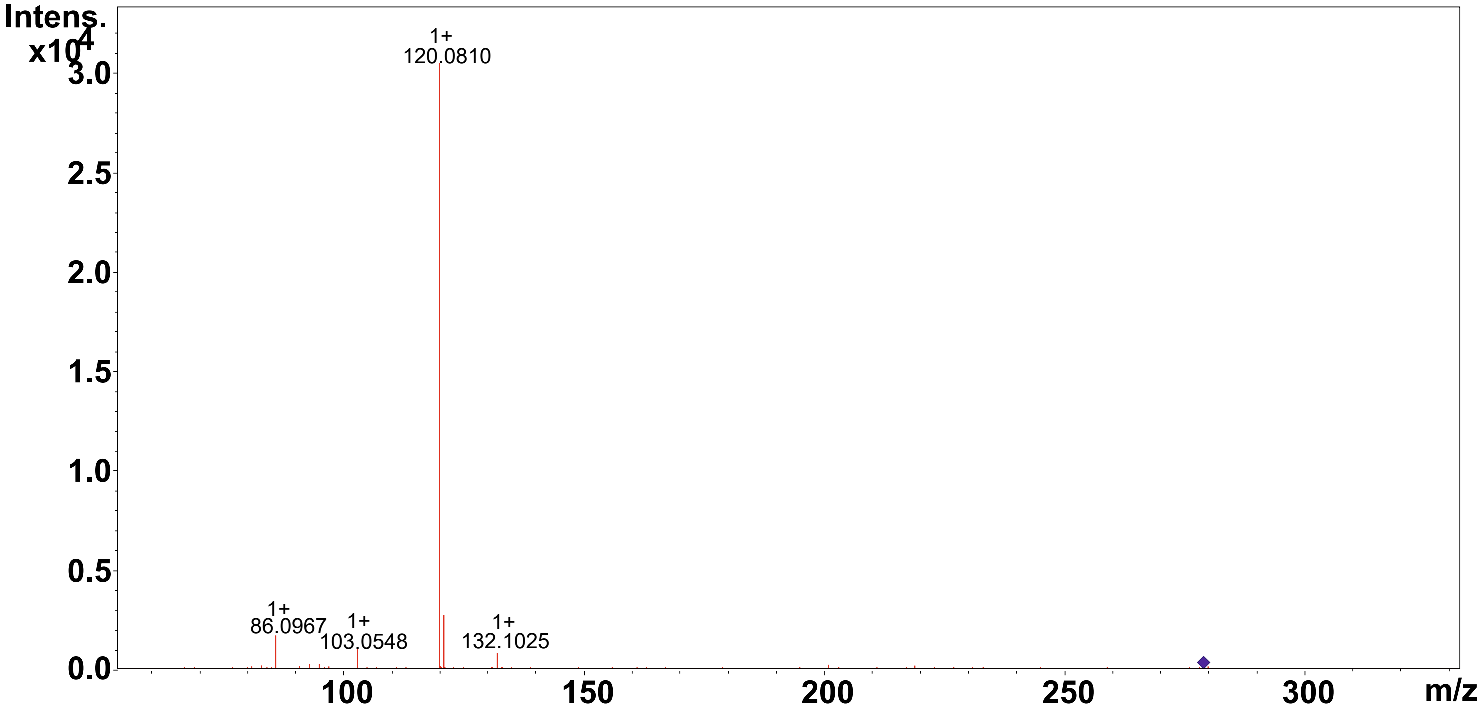

Fig. S11

Compound 9, m/z 263.1281 [M+H]$^+$ and 285.1335 [M+Na]$^+$, 15.5 mins

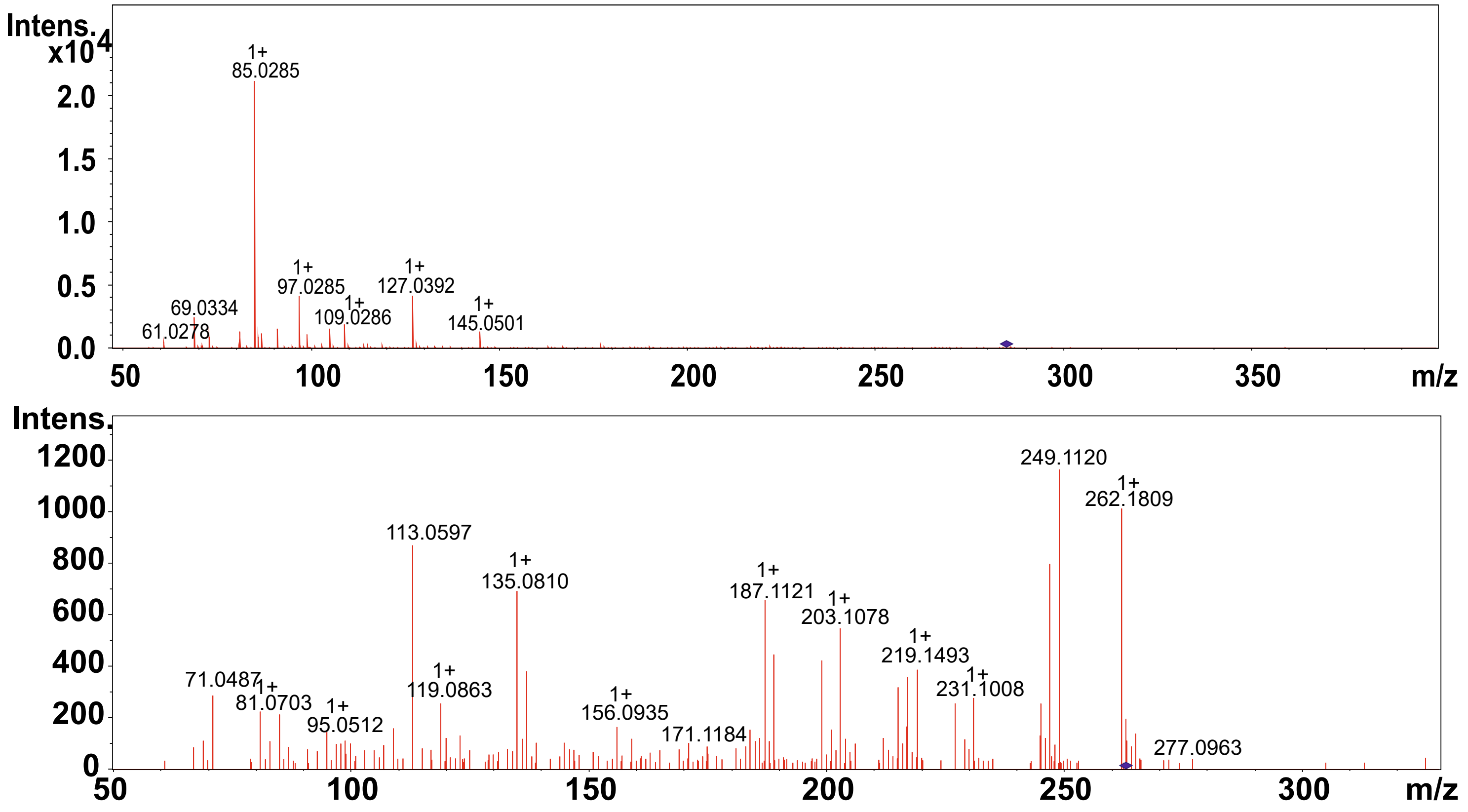

Fig. S12

Compound 10, m/z 343.1179 [M+H]$^+$, 15.6 mins

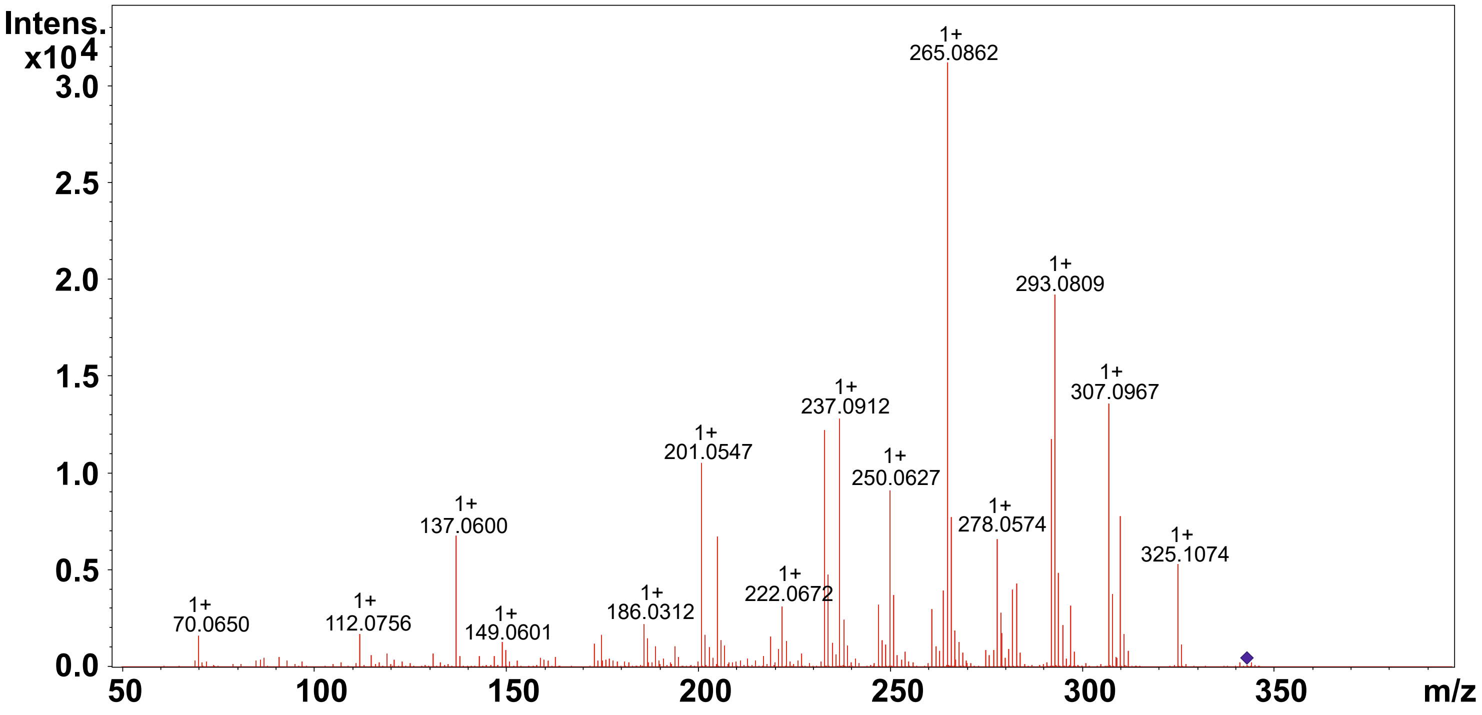

Fig. S13

Compound 11, m/z 285.2215 $[M+H]^+$, 18.3 mins

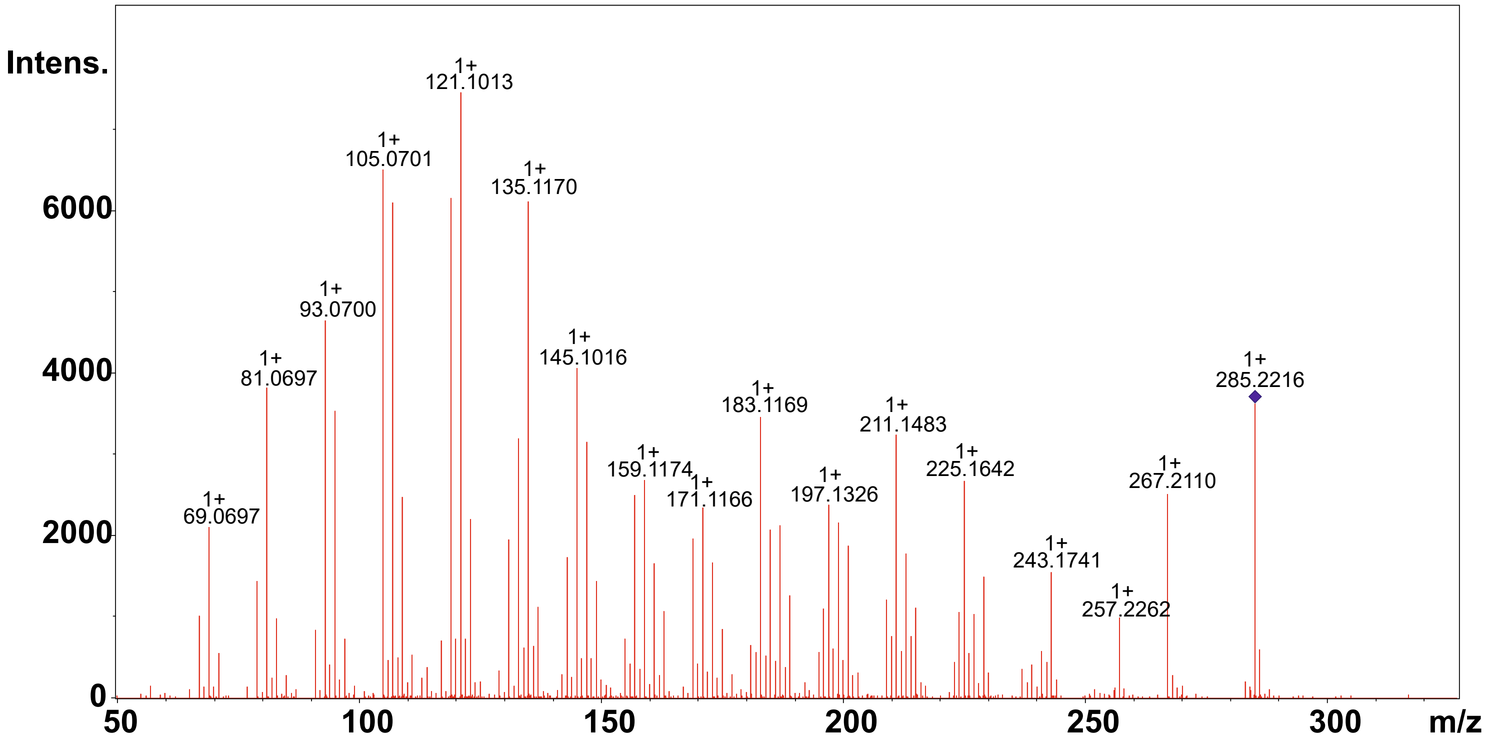

Fig. S14

Compound 12, m/z 227.1281 $[M+H]^+$, 15.9 mins

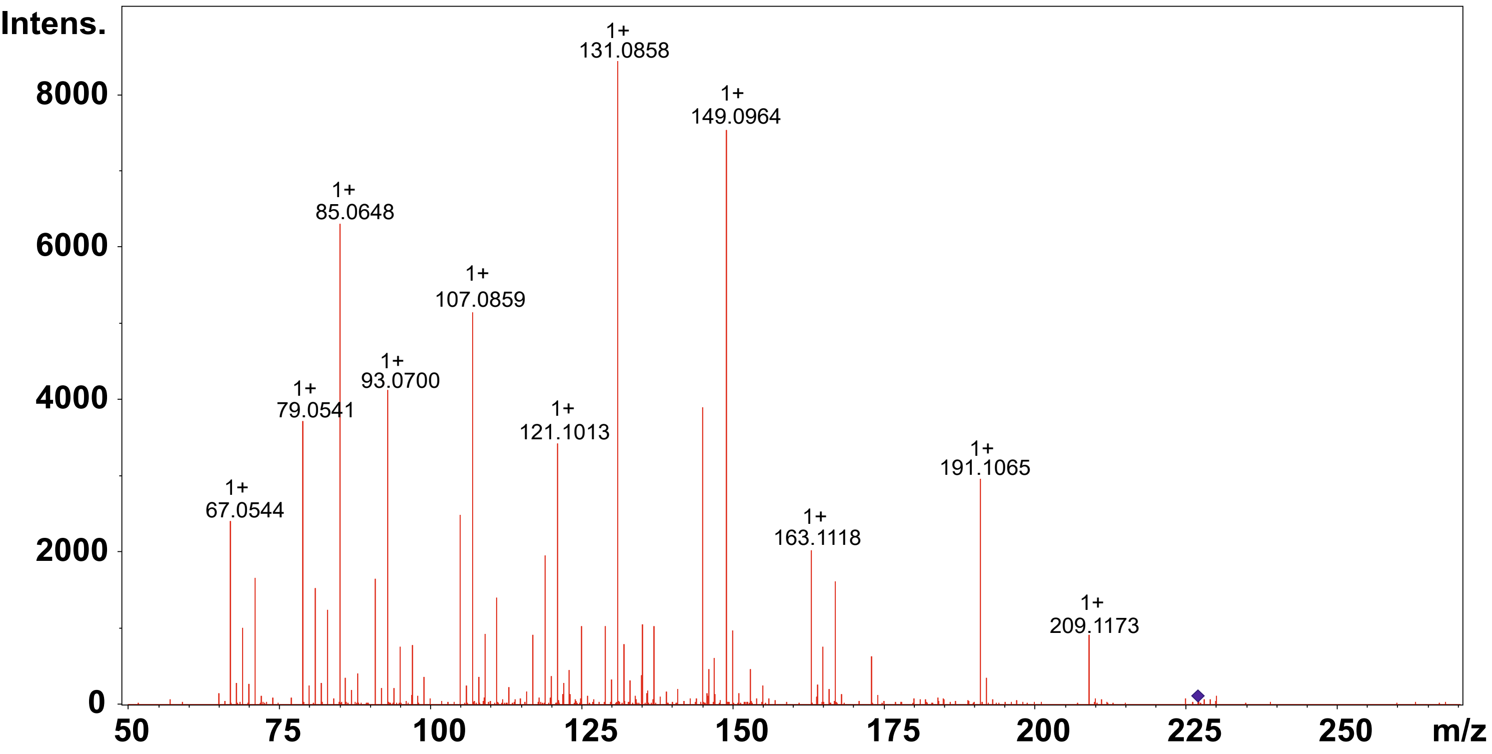

Fig. S15

Compound 13, m/z 339.1054 [M+Na]$^+$, 9.9 mins

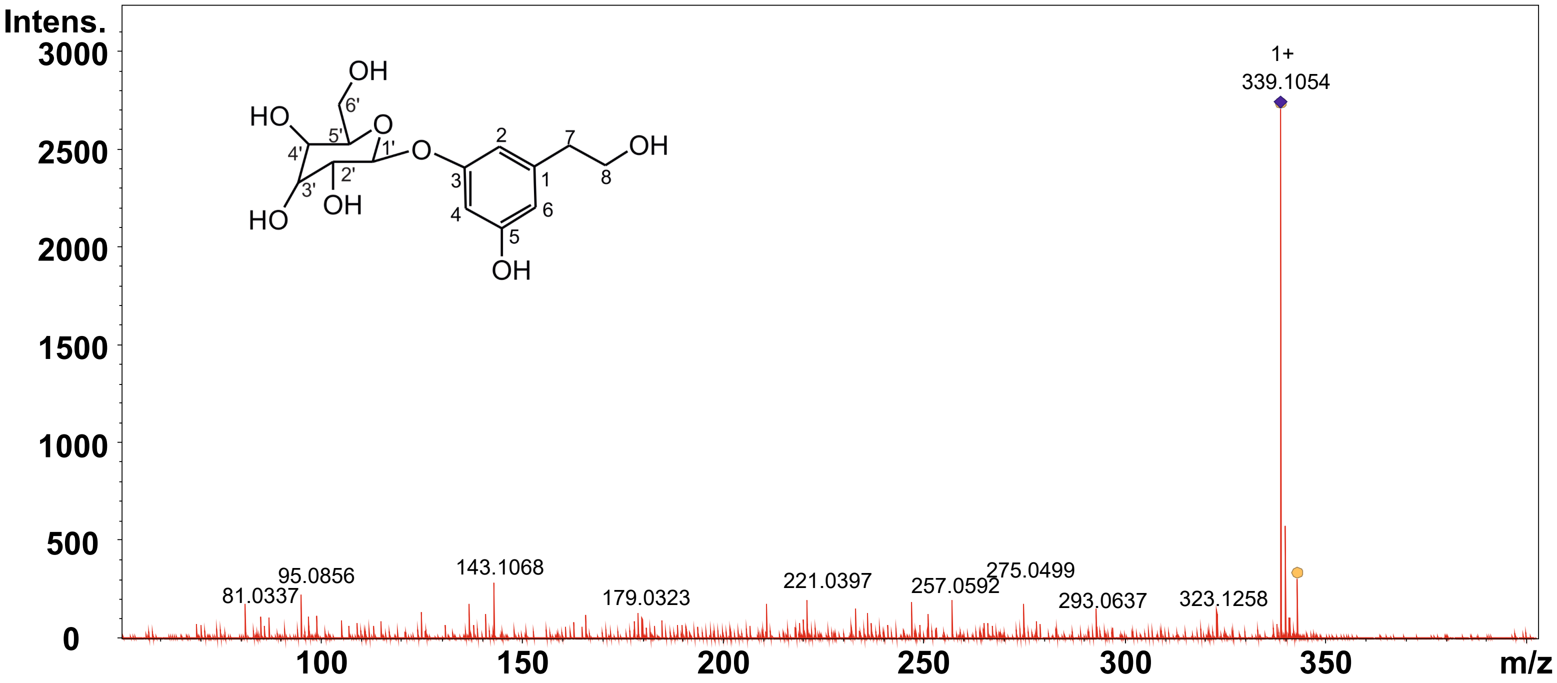

Fig. S16

Compound 14, m/z 341.1384 [M+H]$^+$, 15.1 mins

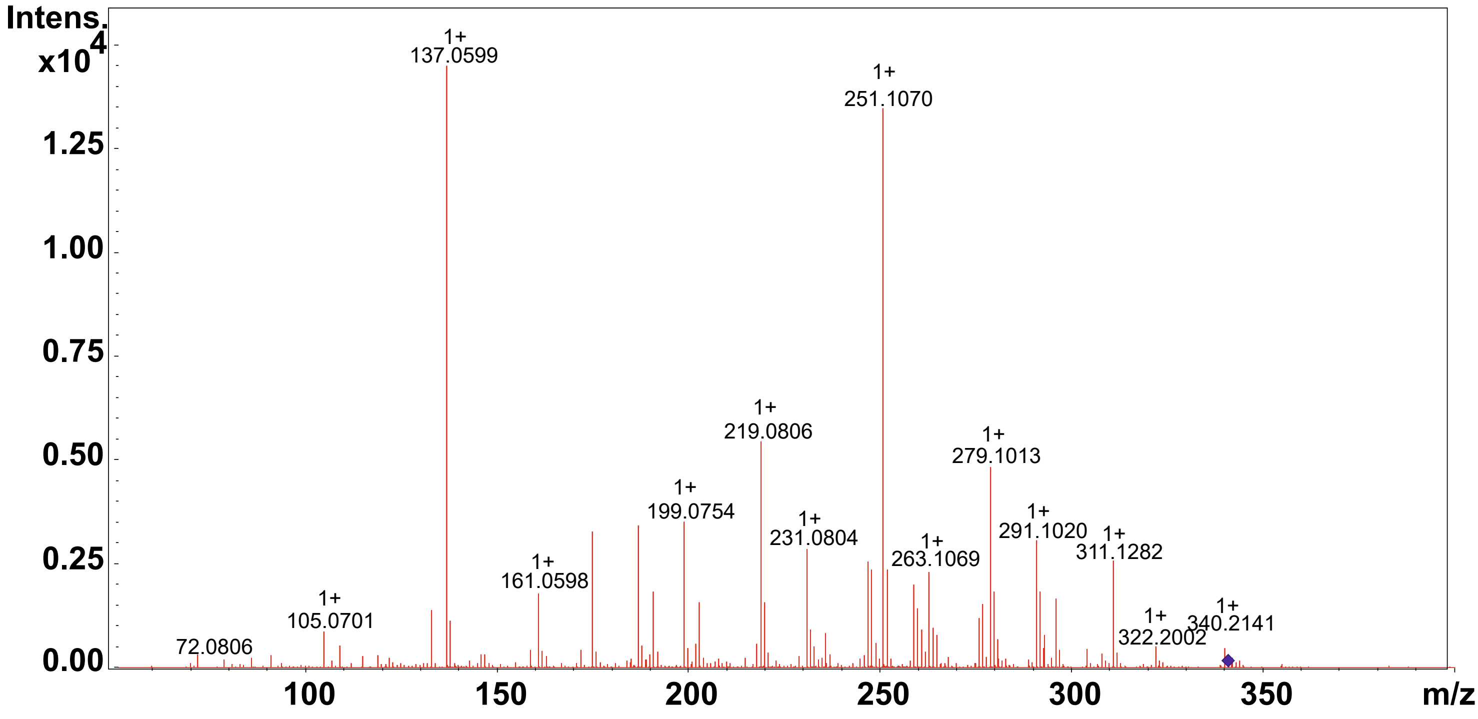

Fig. S17

Compound 15, 322.2018 [M+H]$^+$, 15.8 mins

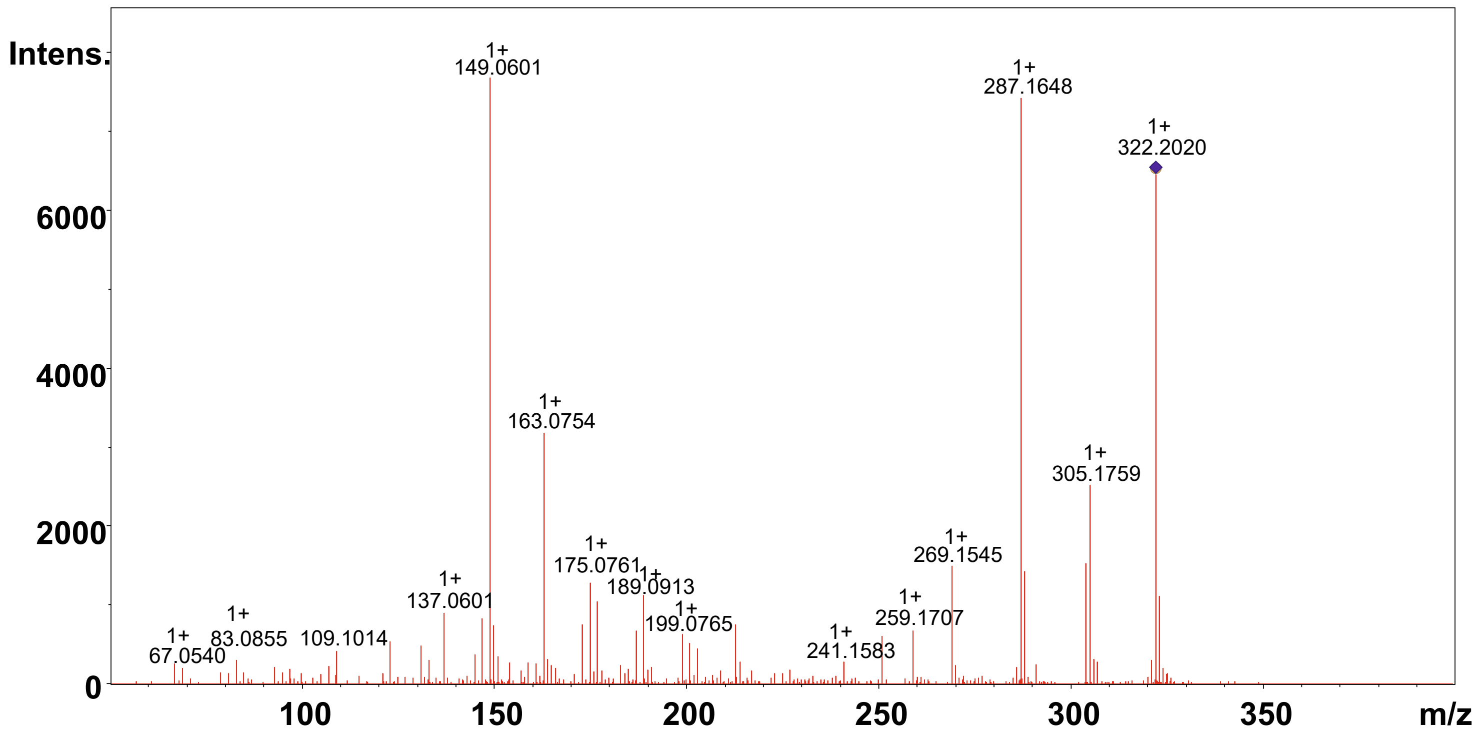

Fig. S18

Compound 16, 536.1762 [M+H]+, 15.0 mins

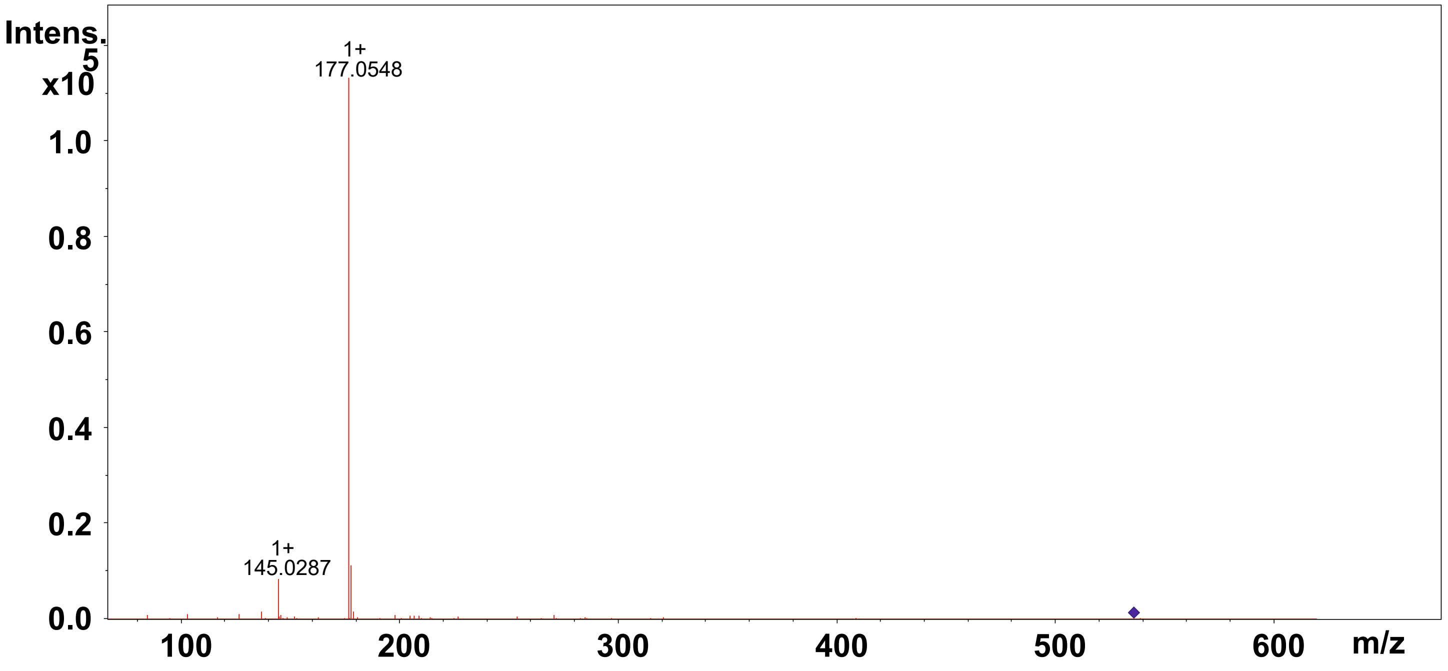

Fig. S19

Compound 17, 743.2035 [M+H]+, 14.2 mins

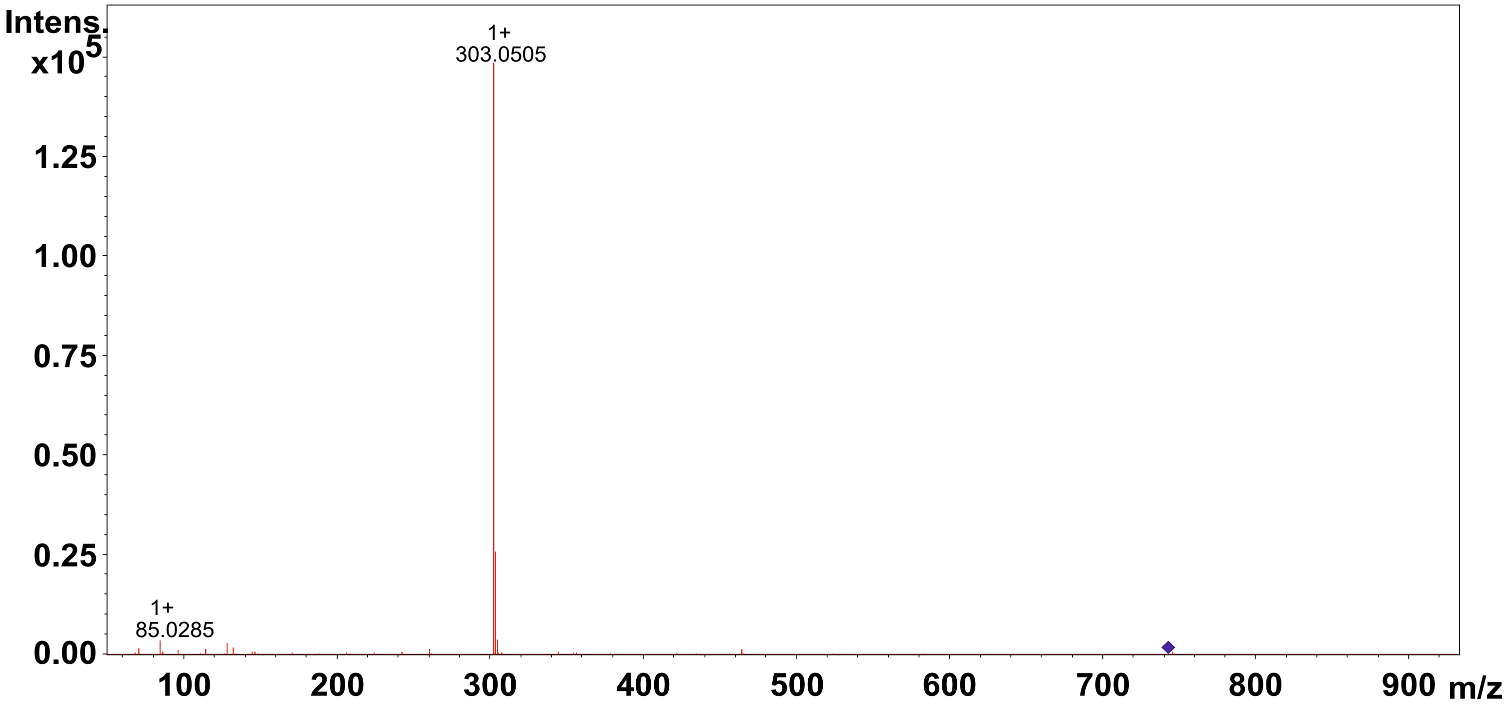

Fig. S20

MS^2 spectra of 8 highly abundant unknown metabolites

Compound 1', m/z 631.3237[M+H]$^+$, 17.2 mins

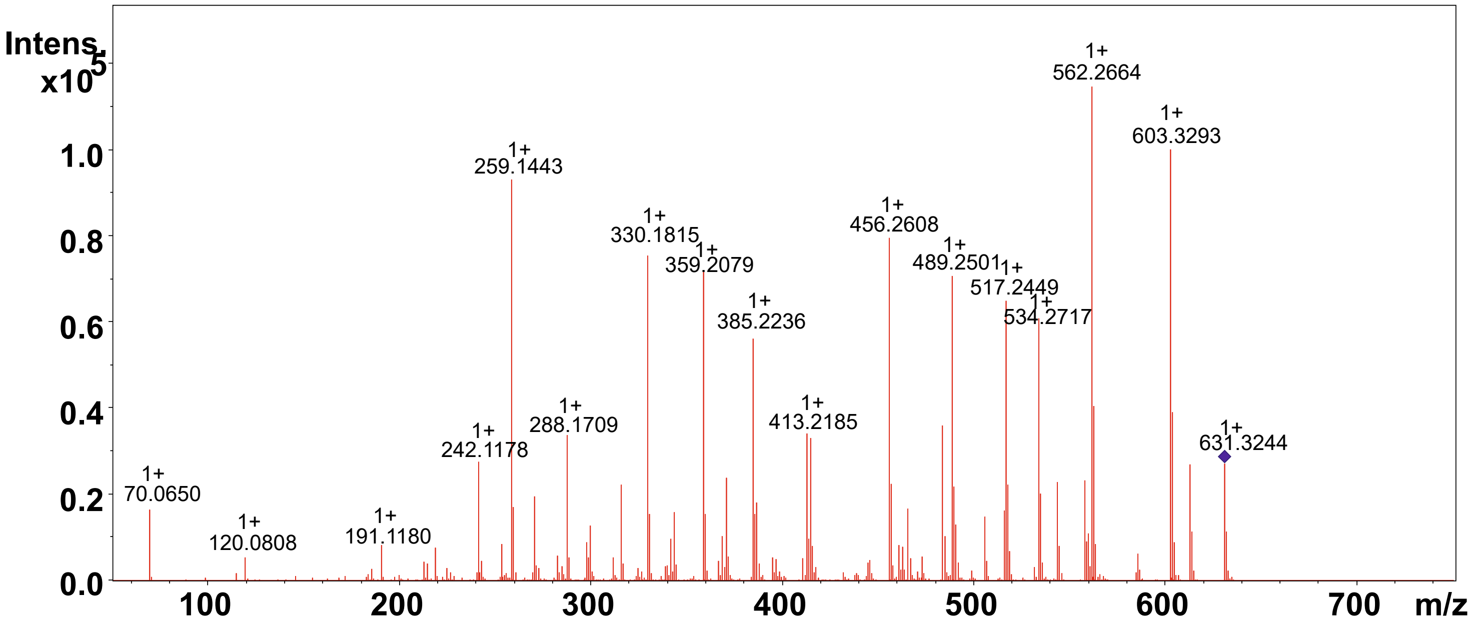

Fig. S21

Compound 2', m/z 671.3555[M+H]$^+$, 21.6 mins

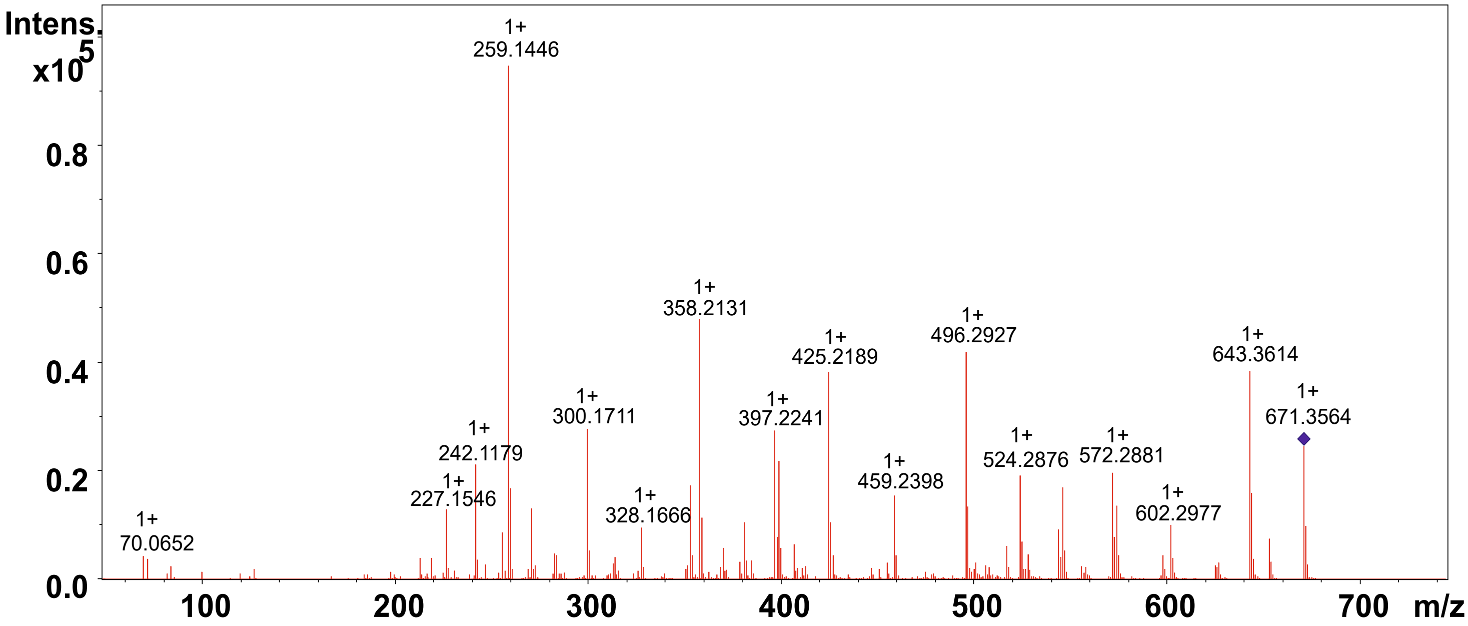

Fig. S22

Compound 3', m/z 673.3709[M+H]$^+$, 20.0 mins

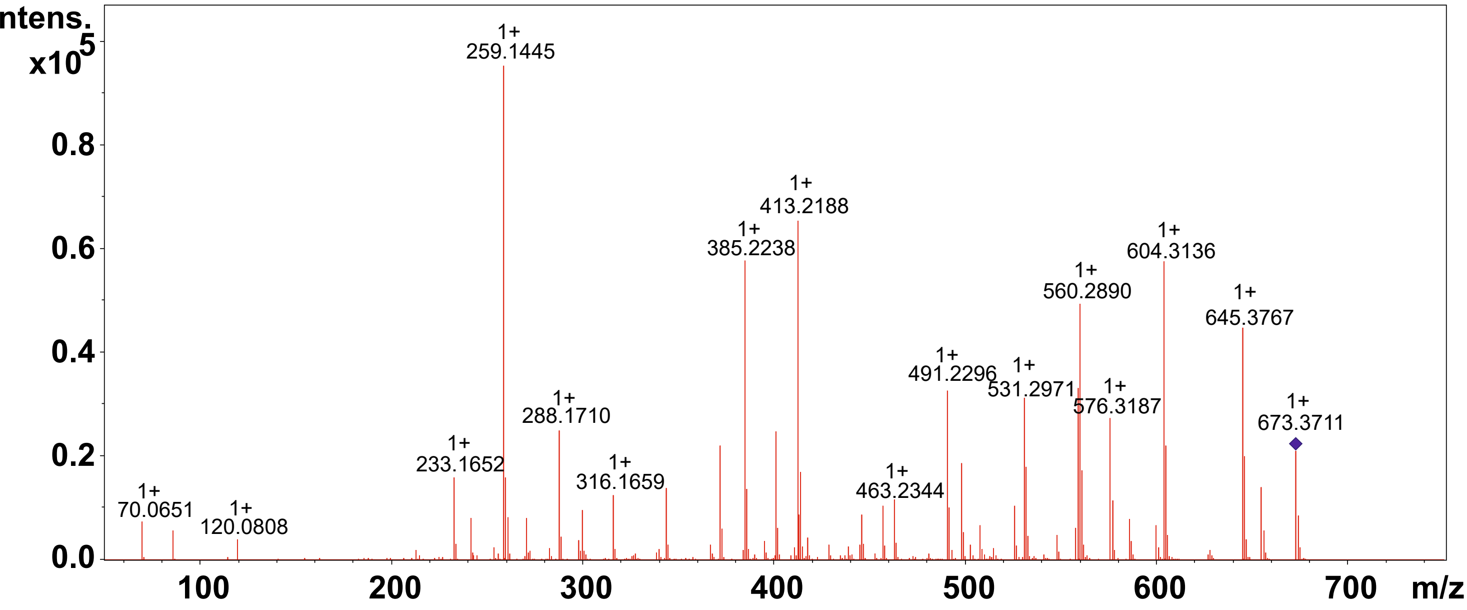

Fig. S23

Compound 4', m/z 550.2863[M+H]$^+$, 15.1 mins

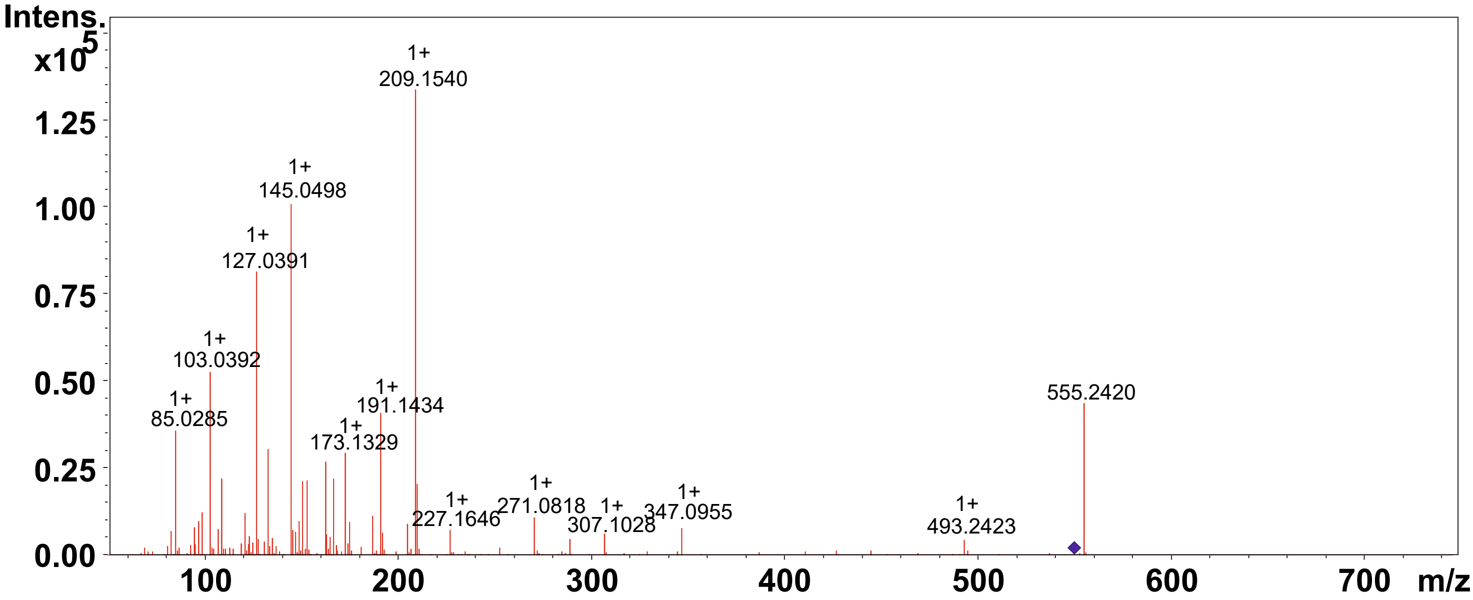

Fig. S24

Compound 5', m/z 490.1973[M+H]$^{+}$, 13.7 mins

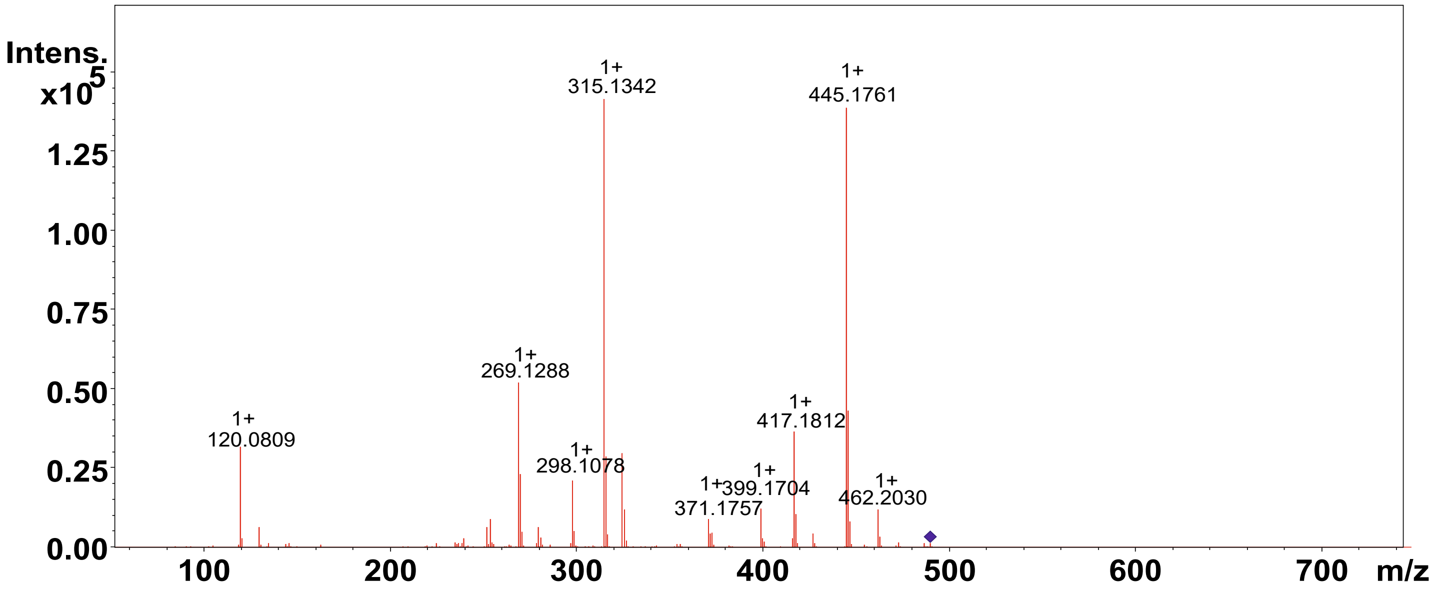

Fig. S25

Compound 6', m/z 432.1713[M+H]$^{+}$, 5.0 mins

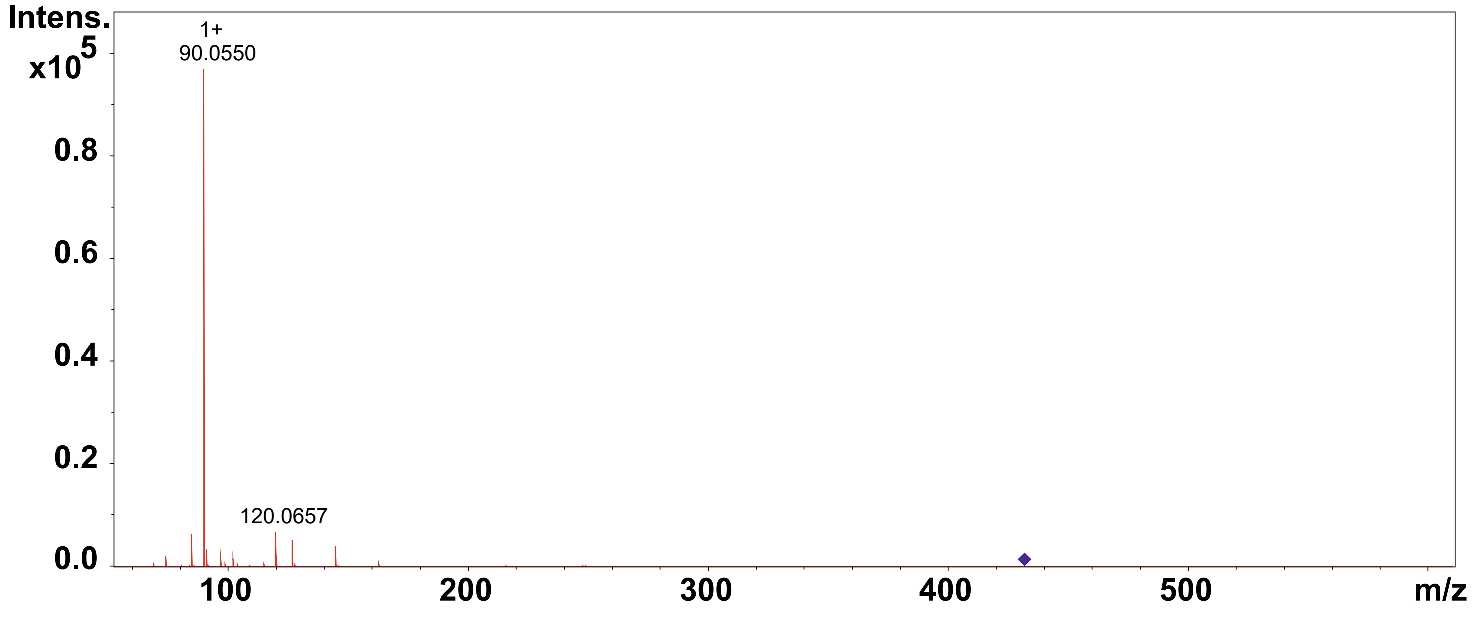

Fig. S26

Compound 7', m/z 446.1874[M+H]$^{+}$, 5.1 mins

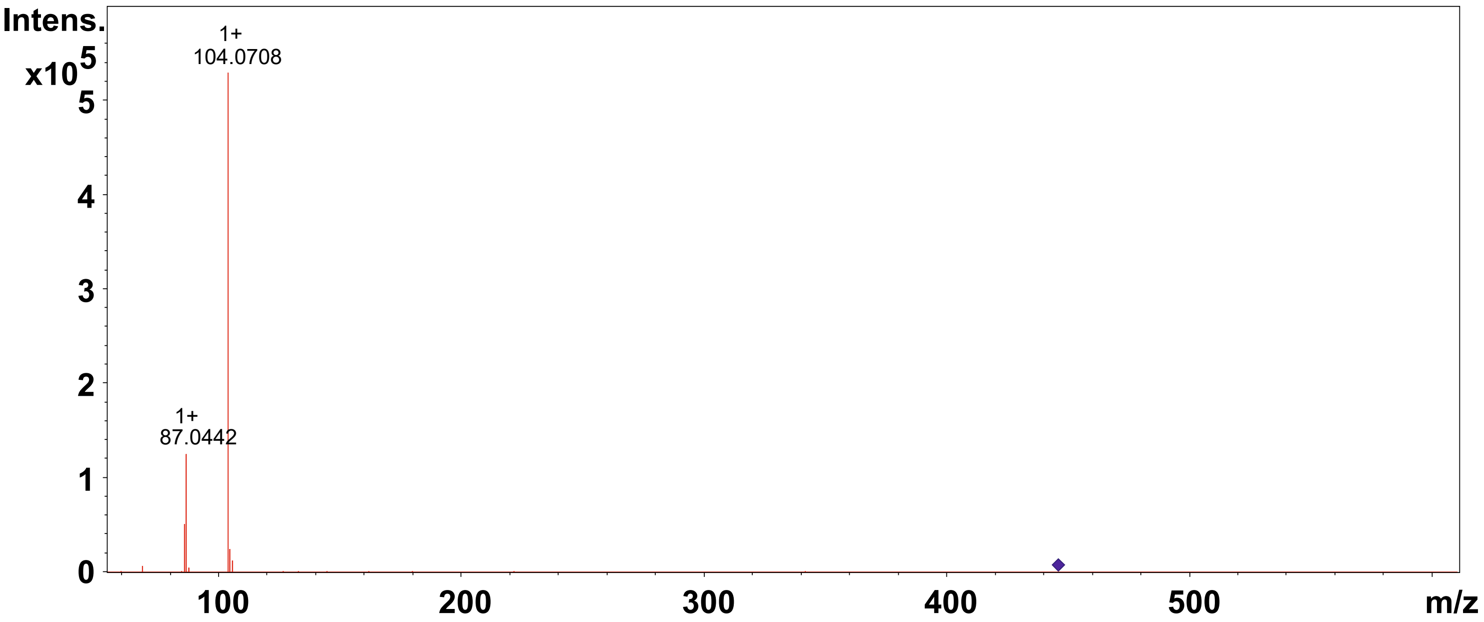

NMR spectra of compound 13 (^{1}H:400 MHz; ^{13}C: 100 MHz, in CD_3OD)

Fig. S27 Spectra 1: HNMR of compound 13

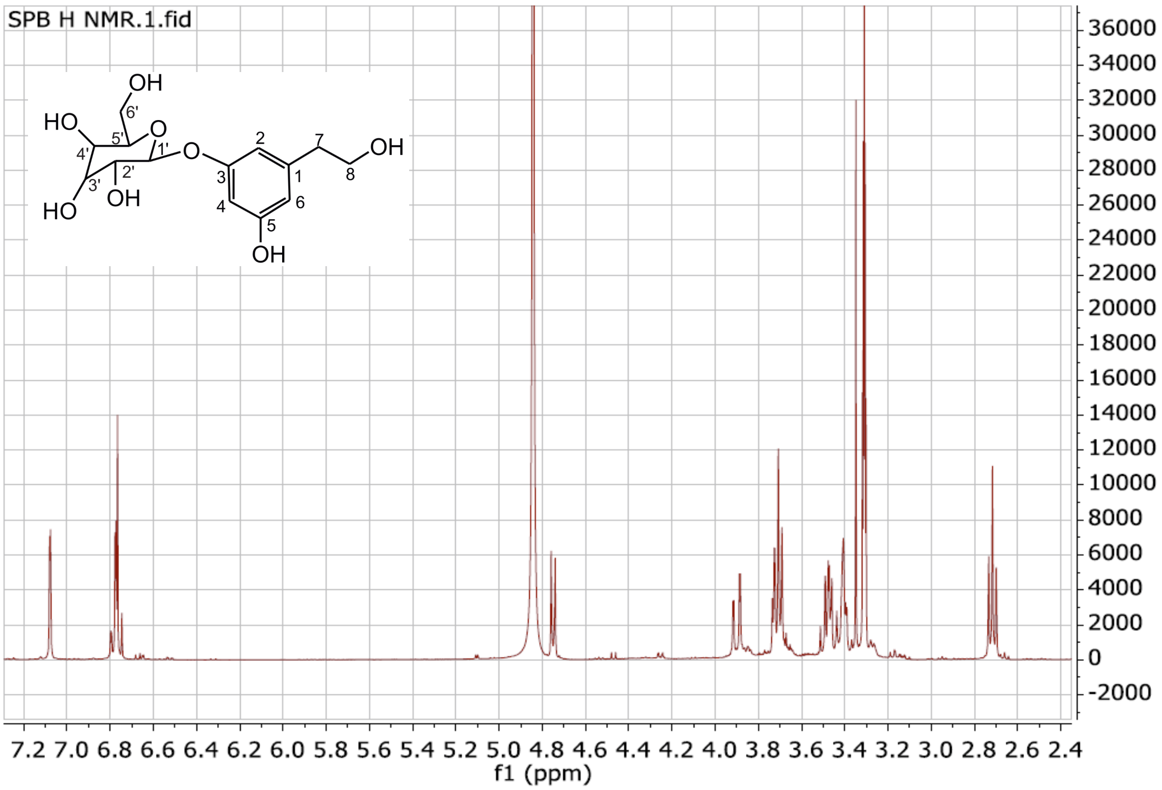

Fig. S28. Spectra 2: ^{13}CNMR of compound 13

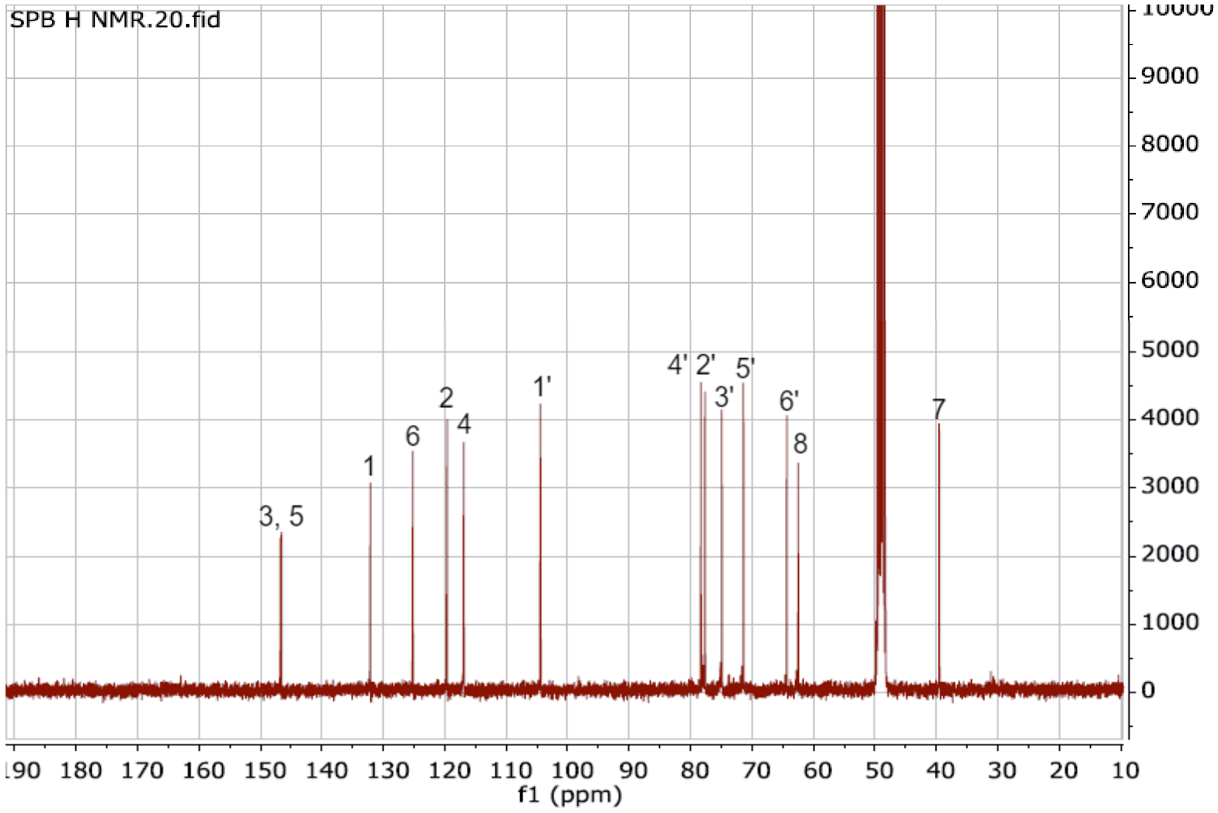

Fig. S29 Spectra 3: DEPT-135 of compound 13

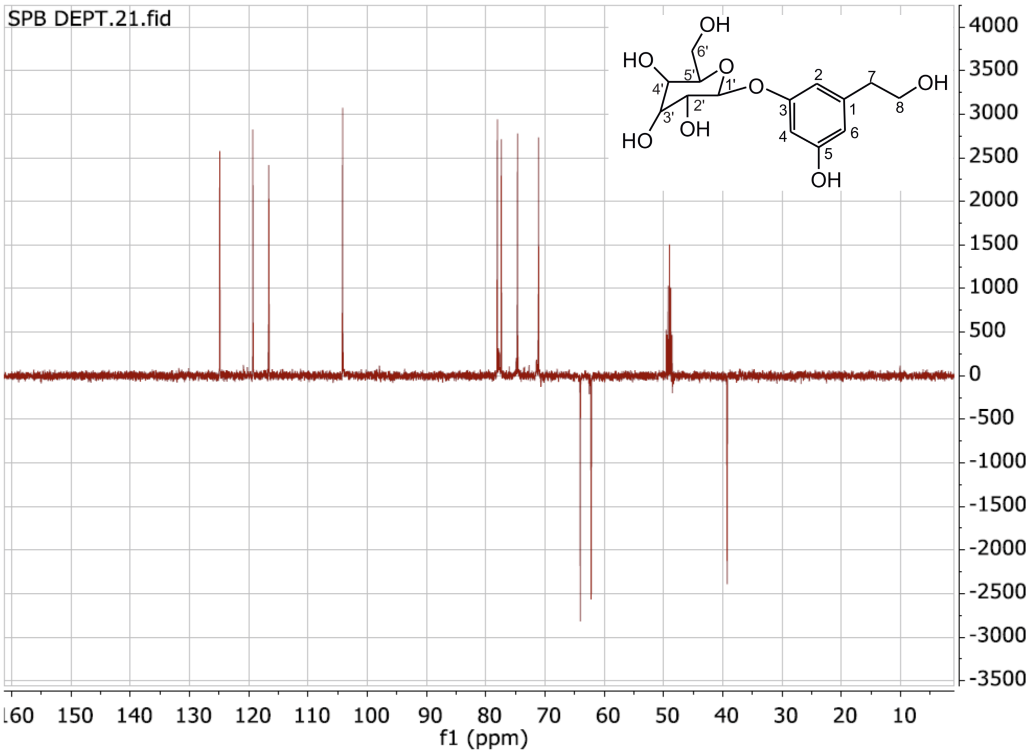

Fig. S30. Spectra 4: COSY of compound 13

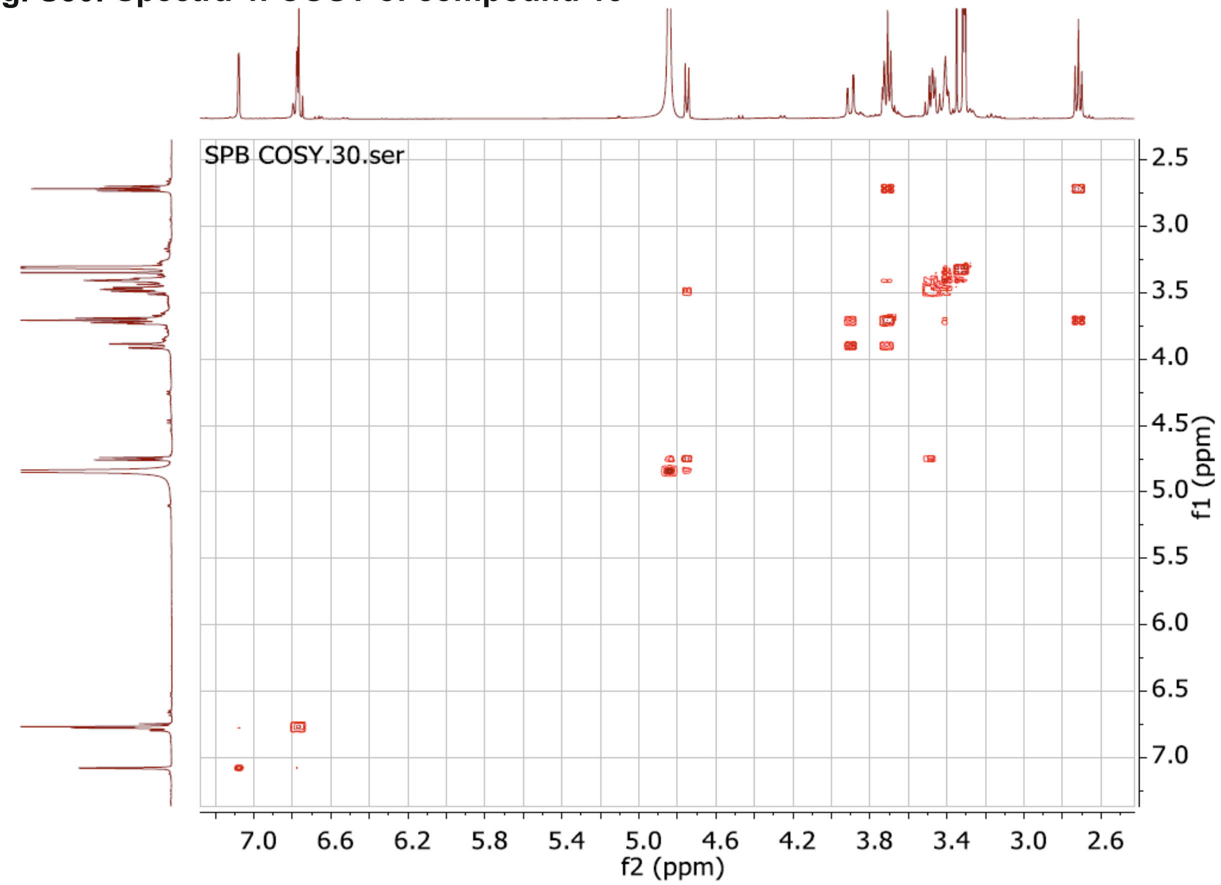

Fig. S31. Spectra 5: HSQC of compound 13

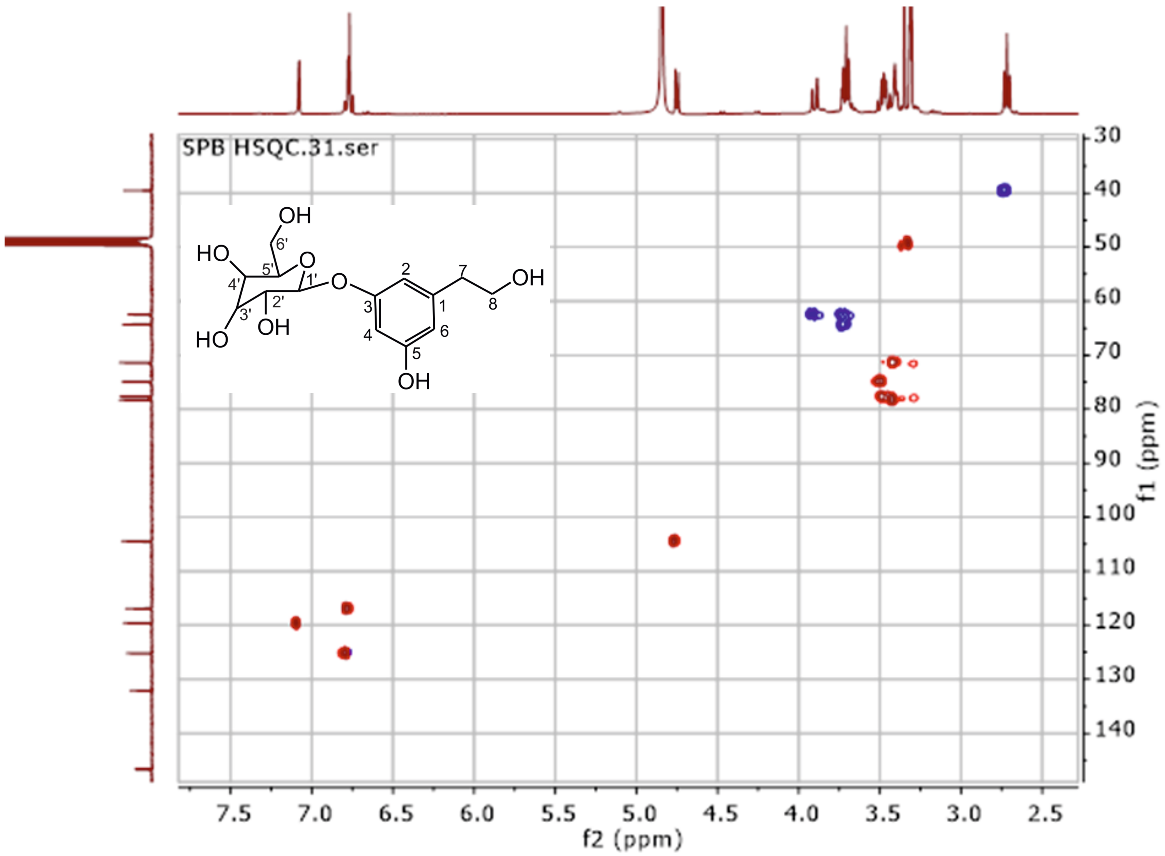

Fig. S32. Spectra 6: HMBC of compound 13

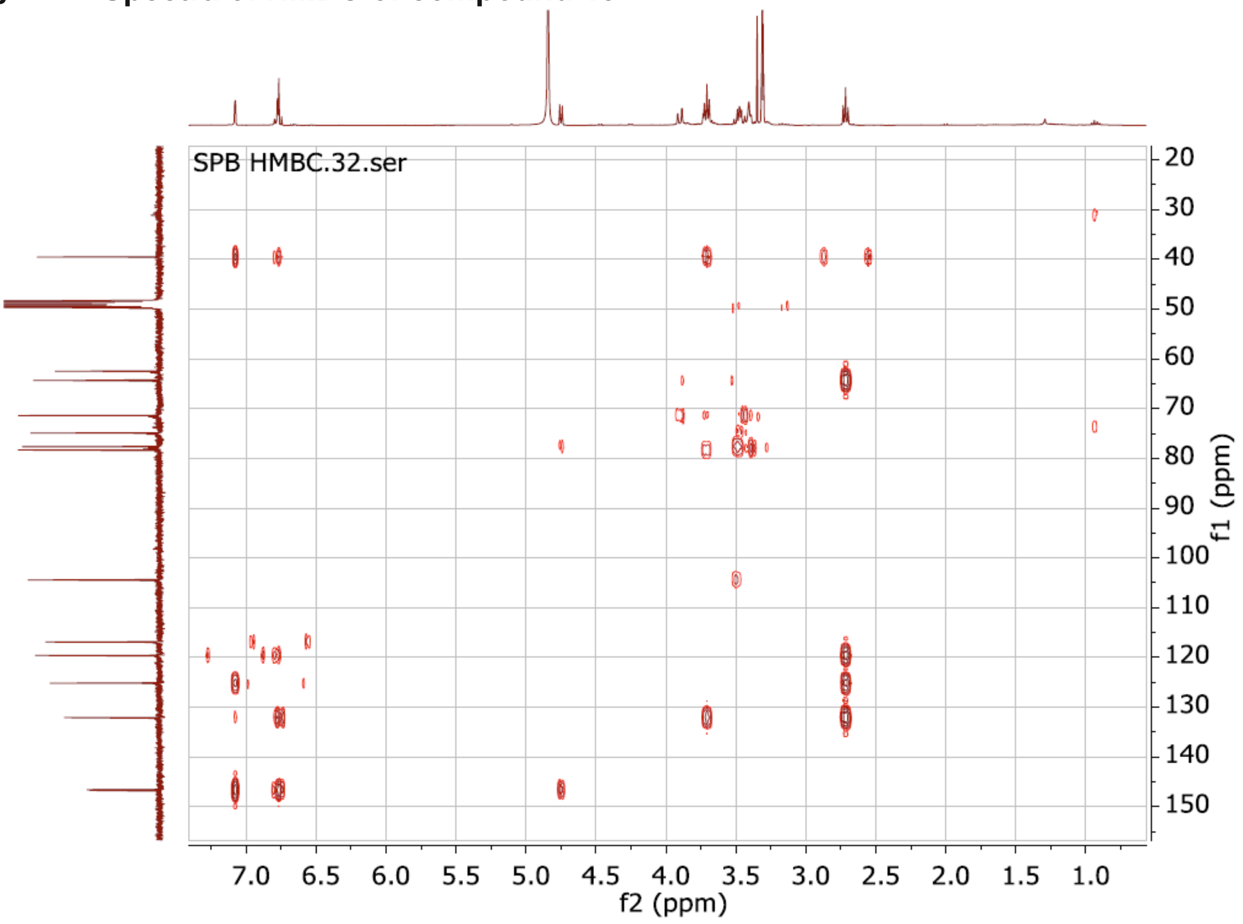

Fig. S33. Fragmentation tree of compound 1'

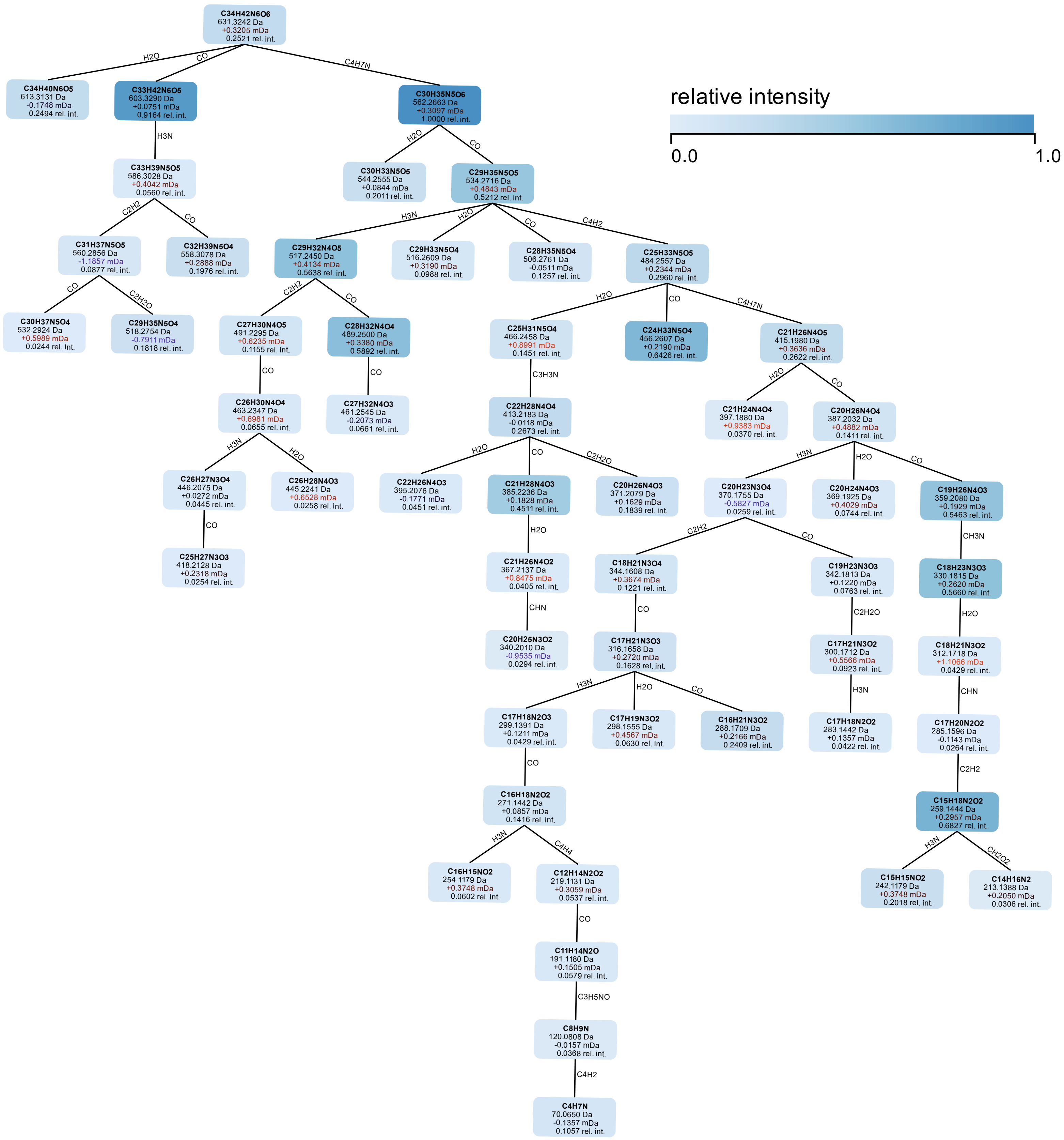

Fig. S34. Fragmentation tree of compound 2'

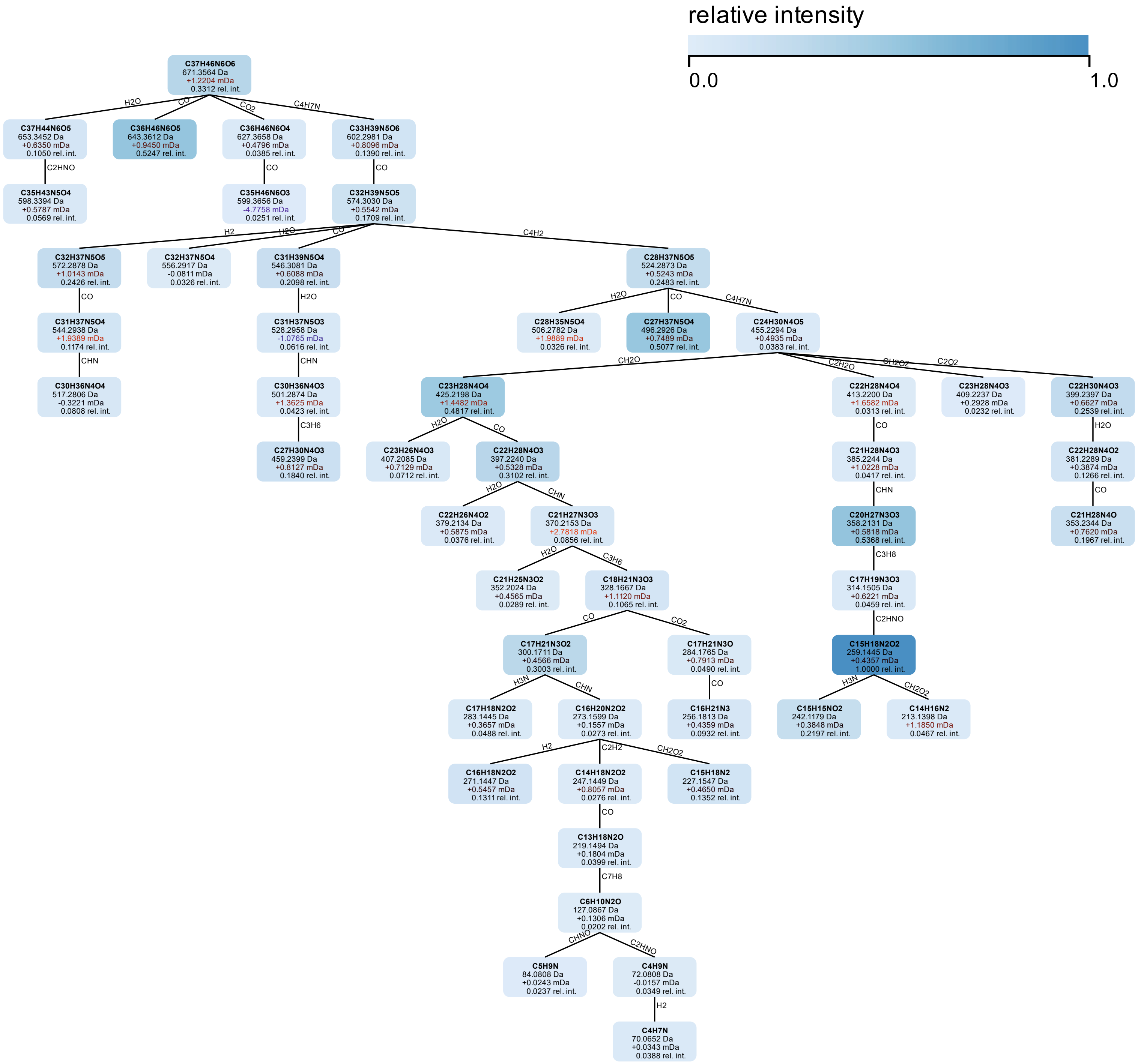

Fig. S35. Fragmentation tree of compound 3'

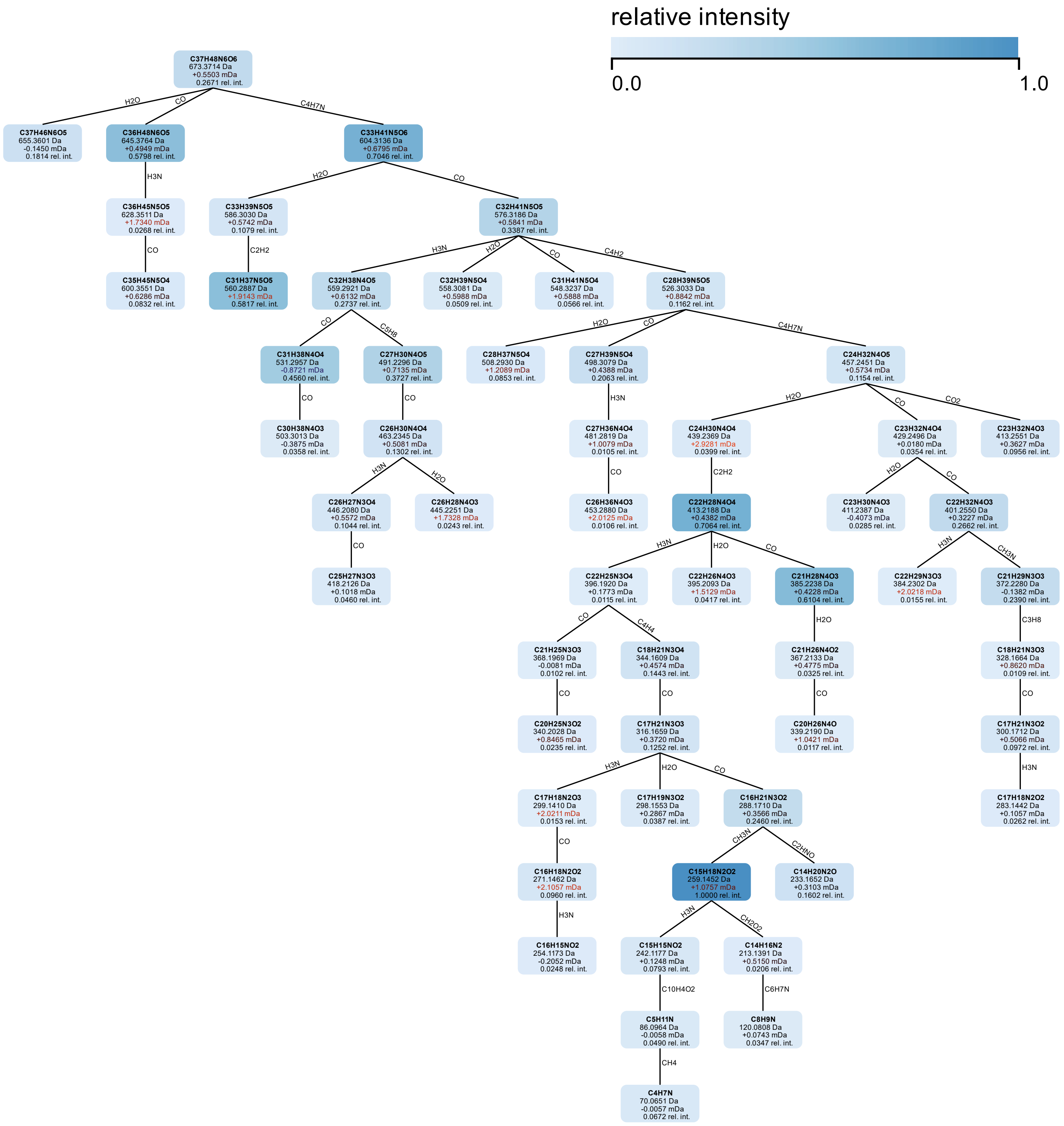

Fig. S36. Fragmentation tree of compound 4'

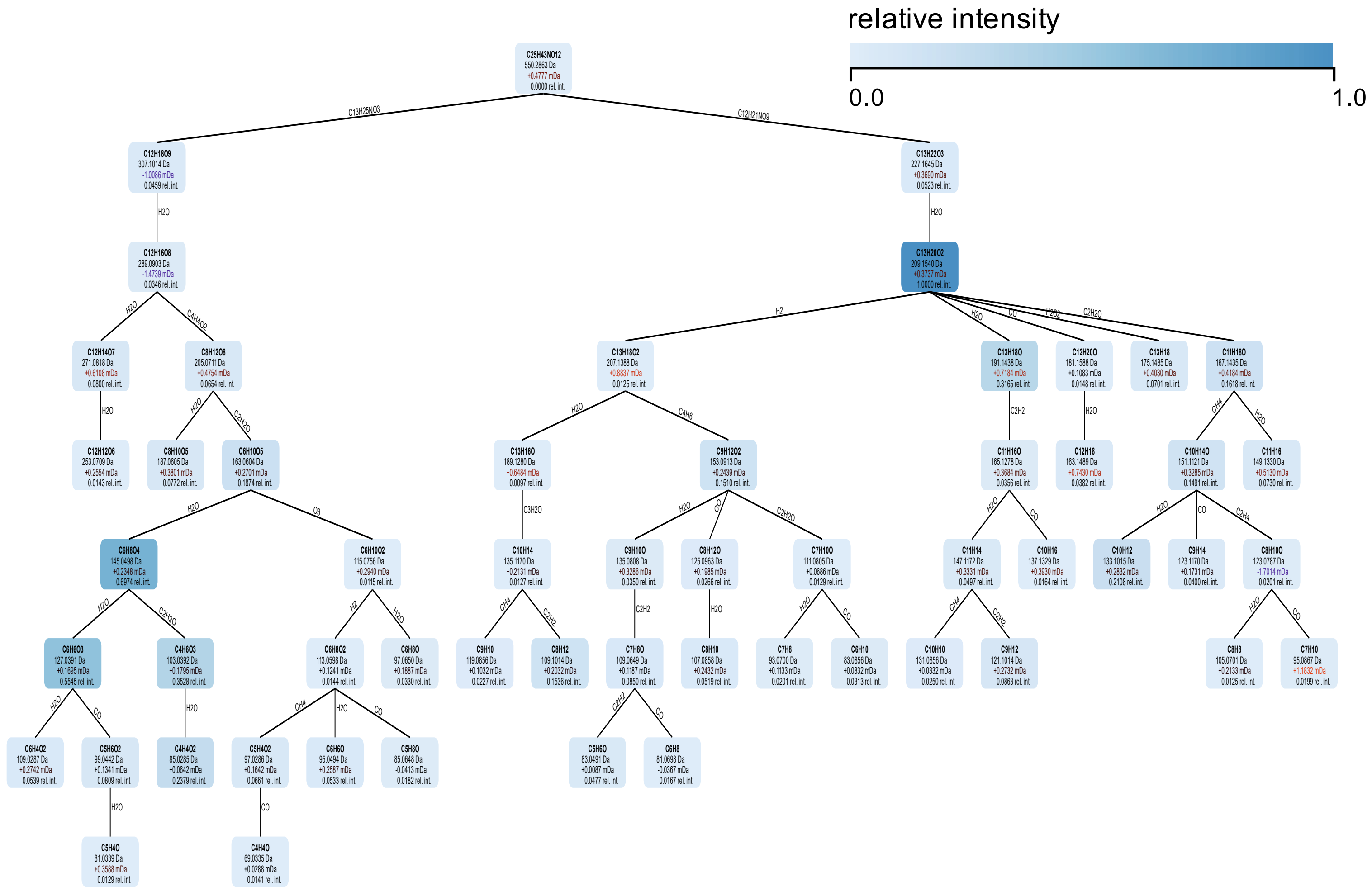

Fig. S37. Fragmentation tree of compound 5'

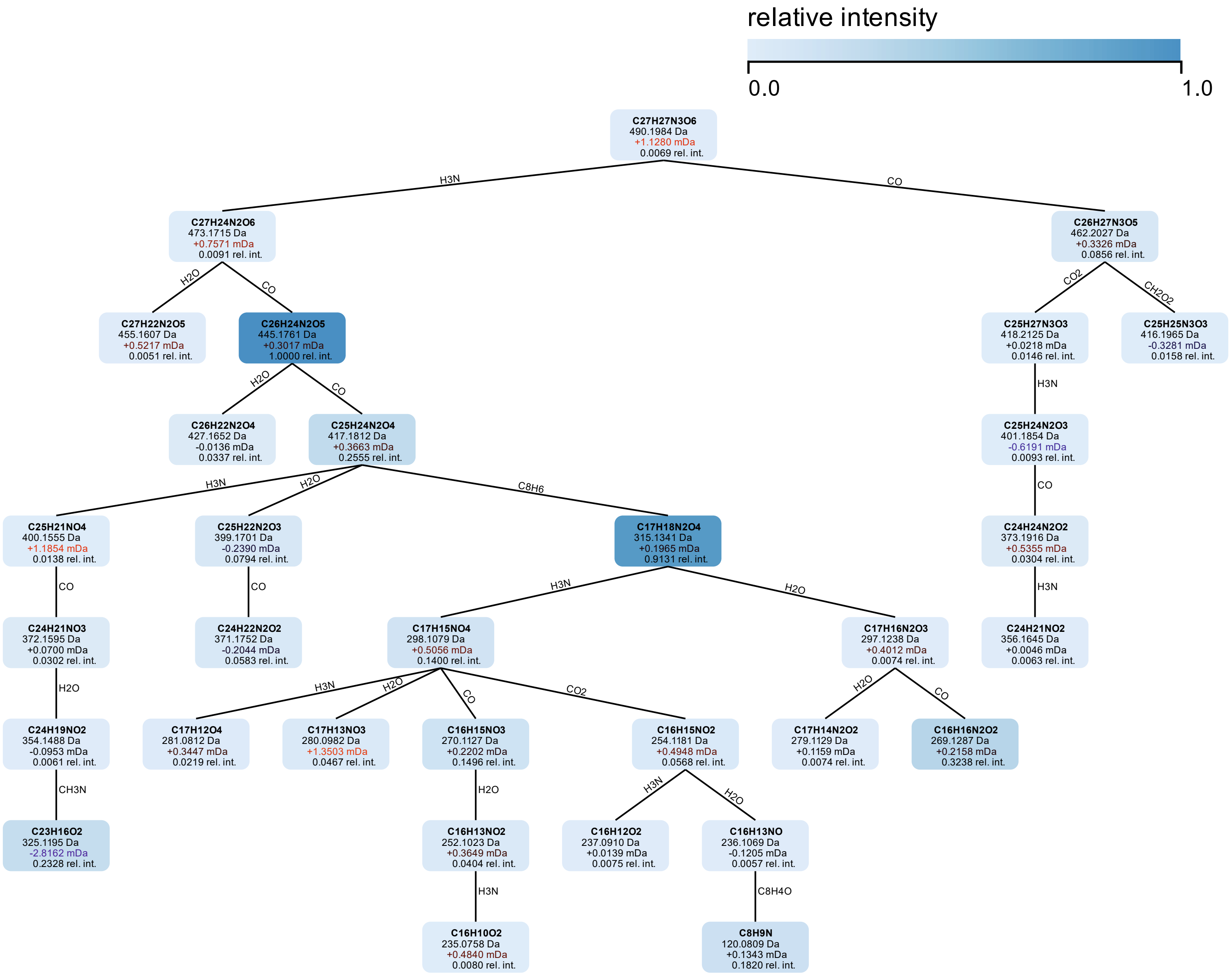

Fig. S38. Fragmentation tree of compound 6'

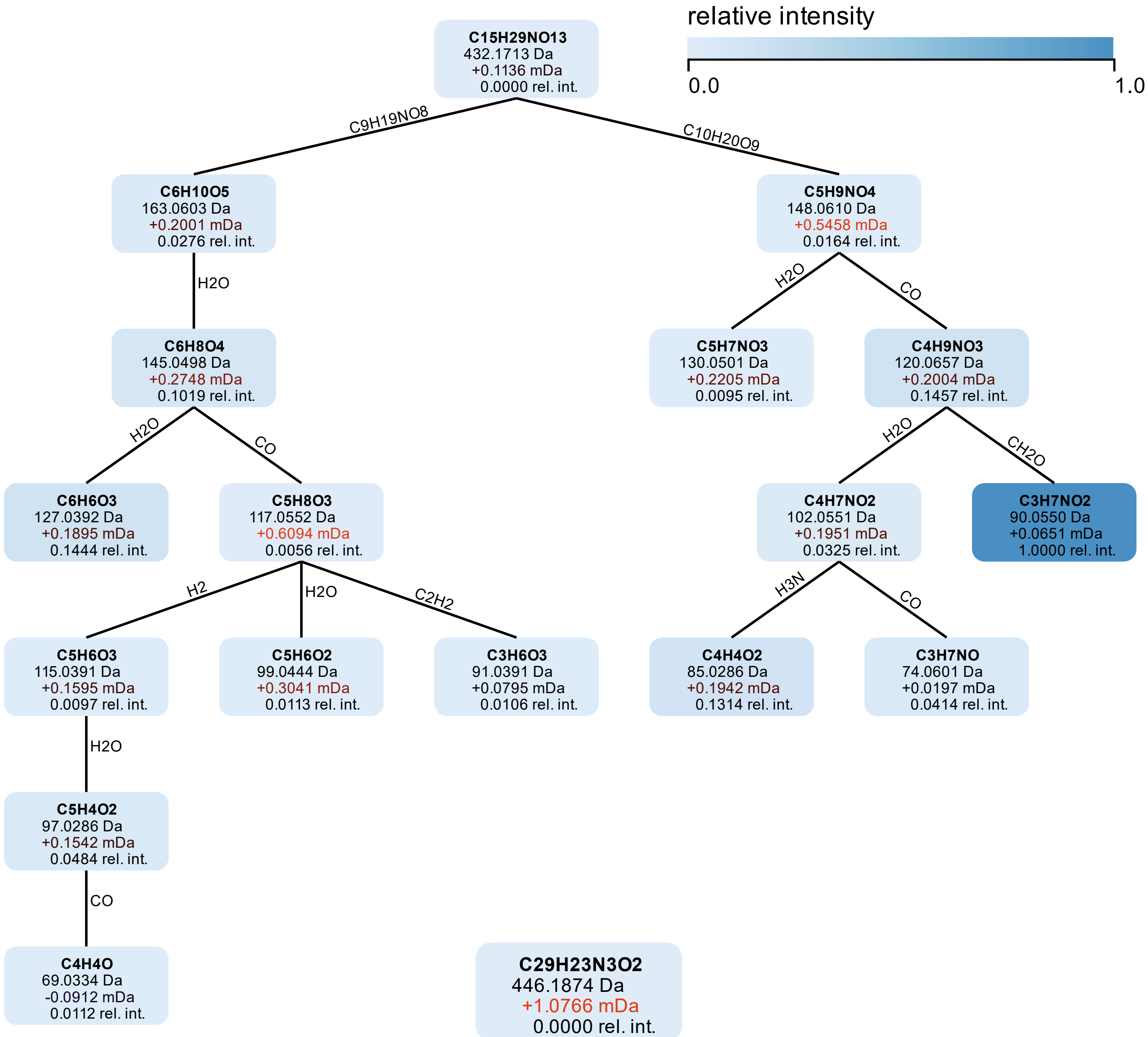

Fig. S39. Fragmentation tree of compound 7'

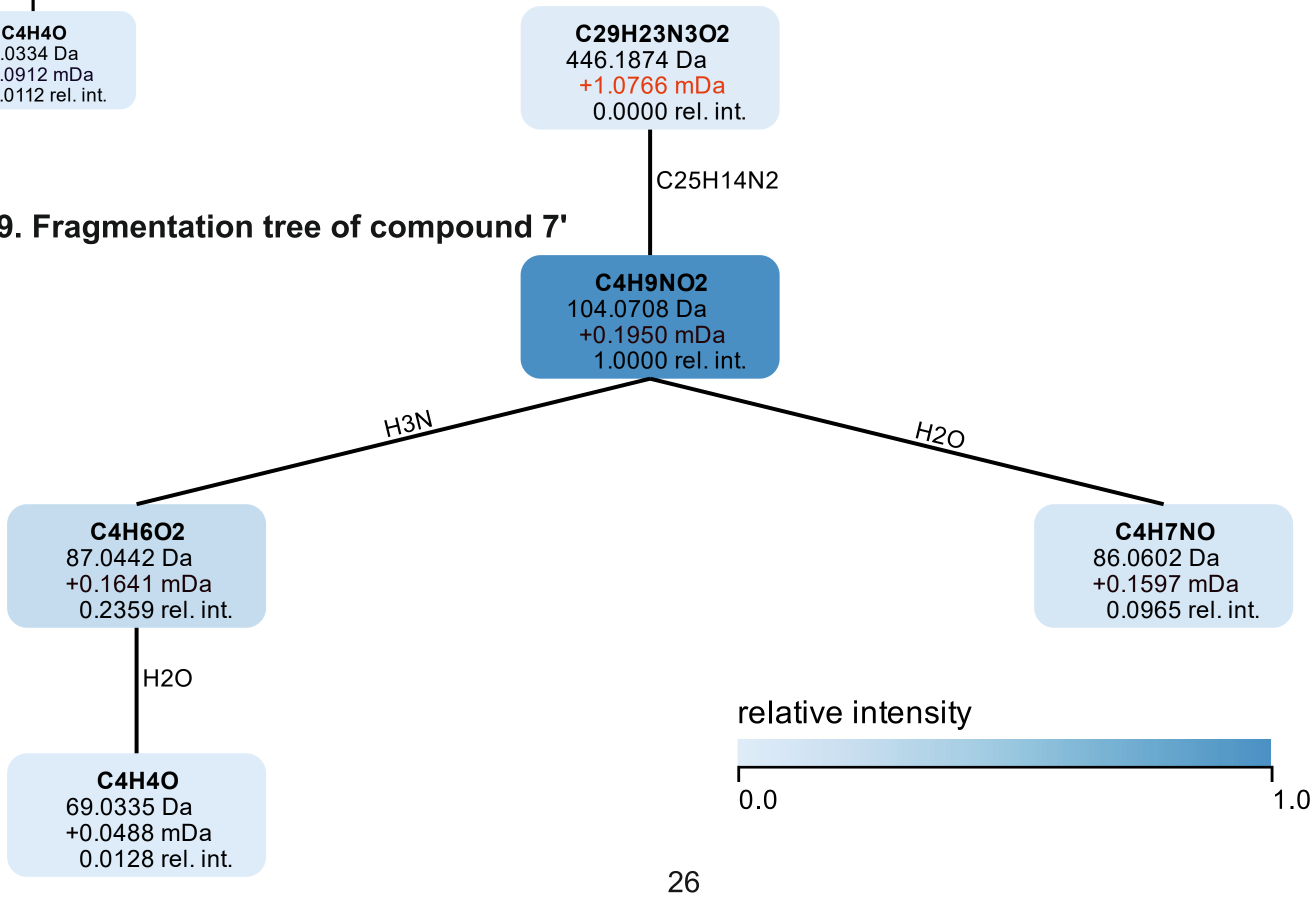

Supplement: Supplementary Materials — The supplementary files for the manuscript are provided in the supporting information attached herewith. [file 3710791.f1.pdf]
